# Habitual sleep disturbances and migraine: a Mendelian randomization study

**DOI:** 10.1002/acn3.51228

**Published:** 2020-10-30

**Authors:** Iyas Daghlas, Angeliki Vgontzas, Yanjun Guo, Daniel I. Chasman, International Headache Genetics Consortium, Padhraig Gormley, Verneri Anttila, Bendik S. Winsvold, Priit Palta, Tonu Esko, Tune H. Pers, Kai‐How Farh, Ester Cuenca‐Leon, Mikko Muona, Nicholas A. Furlotte, Tobias Kurth, Andres Ingason, George McMahon, Lannie Ligthart, Gisela M. Terwindt, Mikko Kallela, Tobias M. Freilinger, Caroline Ran, Scott G. Gordon, Anine H. Stam, Stacy Steinberg, Guntram Borck, Markku Koiranen, Lydia Quaye, Hieab H. H. Adams, Terho Lehtimäki, Antti‐Pekka Sarin, Juho Wedenoja, David A. Hinds, Julie E. Buring, Markus Schürks, Paul M. Ridker, Maria Gudlaug Hrafnsdottir, Hreinn Stefansson, Susan M. Ring, Jouke‐Jan Hottenga, Brenda W. J. H. Penninx, Markus Färkkilä, Ville Artto, Mari Kaunisto, Salli Vepsäläinen, Rainer Malik, Andrew C. Heath, Pamela A. F. Madden, Nicholas G. Martin, Grant W. Montgomery, Mitja I. Kurki, Mart Kals, Reedik Mägi, Kalle Pärn, Eija Hämäläinen, Hailiang Huang, Andrea E. Byrnes, Lude Franke, Jie Huang, Evie Stergiakouli, Phil H. Lee, Cynthia Sandor, Caleb Webber, Zameel Cader, Bertram Muller‐Myhsok, Stefan Schreiber, Thomas Meitinger, Johan G. Eriksson, Veikko Salomaa, Kauko Heikkilä, Elizabeth Loehrer, Andre G. Uitterlinden, Albert Hofman, Cornelia M. van Duijn, Lynn Cherkas, Linda M. Pedersen, Audun Stubhaug, Christopher S. Nielsen, Minna Männikkö, Evelin Mihailov, Lili Milani, Hartmut Göbel, Ann‐Louise Esserlind, Anne Francke Christensen, Thomas Folkmann Hansen, Thomas Werge, Jaakko Kaprio, Arpo J. Aromaa, Olli Raitakari, M. Arfan Ikram, Tim Spector, Marjo‐Riitta Järvelin, Andres Metspalu, Christian Kubisch, David P. Strachan, Michel D. Ferrari, Andrea C. Belin, Martin Dichgans, Maija Wessman, Arn M. J. M. van den Maagdenberg, John‐Anker Zwart, Dorret I. Boomsma, George Davey Smith, Kari Stefansson, Nicholas Eriksson, Mark J. Daly, Benjamin M. Neale, Jes Olesen, Daniel I. Chasman, Dale R. Nyholt, Aarno Palotie, Richa Saxena

**Affiliations:** ^1^ Broad Institute of MIT and Harvard 415 Main Street Cambridge Massachusetts 02142 USA; ^2^ Center for Genomic Medicine Massachusetts General Hospital 185 Cambridge Street Boston Massachusetts 02114 USA; ^3^ Division of Preventive Medicine Department of Medicine Brigham and Women's Hospital Harvard Medical School Boston Massachusetts 02115 USA; ^4^ Department of Neurology Brigham and Women’s Hospital Harvard Medical School Boston Massachusetts 02115 USA; ^5^ Anesthesia, Critical Care and Pain Medicine Massachusetts General Hospital Harvard Medical School Boston Massachusetts 02114 USA

## Abstract

**Objective:**

Sleep disturbances are associated with increased risk of migraine, however the extent of shared underlying biology and the direction of causal relationships between these traits is unclear. Delineating causality between sleep patterns and migraine may offer new pathophysiologic insights and inform subsequent intervention studies. Here, we used genetic approaches to test for shared genetic influences between sleep patterns and migraine, and to test whether habitual sleep patterns may be causal risk factors for migraine and vice versa.

**Methods:**

To quantify genetic overlap, we performed genome‐wide genetic correlation analyses using genome‐wide association studies of nine sleep traits in the UK Biobank (*n* ≥ 237,627), and migraine from the International Headache Genetics Consortium (59,674 cases and 316,078 controls). We then tested for potential causal effects between sleep traits and migraine using bidirectional, two‐sample Mendelian randomization.

**Results:**

Seven sleep traits demonstrated genetic overlap with migraine, including insomnia symptoms (rg = 0.29, *P* < 10^−31^) and difficulty awakening (rg = 0.11, *P* < 10^−4^). Mendelian randomization analyses provided evidence for potential causal effects of difficulty awakening on risk of migraine (OR [95% CI] = 1.37 [1.12–1.68], *P* = 0.002), and nominal evidence that liability to insomnia symptoms increased the risk of migraine (1.09 [1.02–1.16], *P* = 0.02). In contrast, there was minimal evidence for an effect of migraine liability on sleep patterns or disturbances.

**Interpretation:**

These data support a shared genetic basis between several sleep traits and migraine, and support potential causal effects of difficulty awakening and insomnia symptoms on migraine risk. Treatment of sleep disturbances may therefore be a promising clinical intervention in the management of migraine.

## Introduction

Migraine is a debilitating and highly prevalent chronic pain condition that is a leading contributor to disability worldwide.^1^ By the time of clinical presentation, those with migraine are more likely to report several comorbidities, including several sleep disturbances and disorders (reviewed by Vgontzas and Pavlović).^2^, ^3^, ^4^, ^5^, ^6^ Prospective studies have found associations between insomnia and increased risk for incident migraine diagnosis^7^ and vice versa. Despite this epidemiologic evidence, there remain several unanswered questions about the relationship between migraine and sleep. Although both migraine (SNP‐based heritability 15%)^8^ and sleep traits (SNP‐based heritability ranging from 6.9% to 17%)^9^, ^10^, ^11^, ^12^ are heritable, it is unknown whether this comorbidity is driven, at least partly, by shared genetic influences. It is also unknown whether causality underlies this comorbidity,^4^ as associations in epidemiologic studies are potentially biased by residual confounding and reverse causality. Delineating causality between sleep patterns and migraine may offer new pathophysiologic insights into these traits and inform subsequent intervention trials.

Causality can be investigated using Mendelian randomization (MR).^13^ MR can be conceptualized as a natural experiment whereby individuals are randomly allocated to lifelong greater exposure to a given risk factor (e.g., insomnia symptoms) based on their genetic risk, and then the risk of a disease outcome (e.g., migraine) as a function of this exposure is measured later in life.^14^ The validity of this approach rests on the random assortment of genetic alleles at gametogenesis, thereby rendering the alleles relatively unconfounded by environmental factors. Moreover, inherited genetic variation is fixed at birth and is therefore not modifiable by environmental factors or disease status. MR has been previously used to examine causal relationships between migraine and dementia,^15^ blood pressure,^16^ and cardiovascular disease.^17^, ^18^

The availability of large‐scale genome‐wide association studies (GWAS) for sleep traits^9^, ^10^, ^11^, ^12^ (*n ≤ *452,071) and migraine^8^ (*n* = 375,752) now provides an opportunity to test shared genetic predisposition and causal effects. Here, we leveraged cross‐trait LD Score regression^19^ and MR^20^ using recently available data from the UK Biobank cohort and the largest GWAS of migraine^8^ to, respectively, assess for a shared genetic basis and for potential causal effects between sleep traits and migraine.

## Methods

### Data sources: sleep GWAS

#### Sleep traits in UK Biobank

Genetic associations for sleep traits were obtained from published^9^, ^10^, ^12^, ^21^ and unpublished GWAS summary statistics in UK Biobank (UKB) participants of European ancestry (methodologic details given in Data S1; GWAS characteristics listed in Table S1). We considered GWAS for all sleep traits ascertained in UKB: sleep duration,^12^ morning diurnal preference (also referred to as “chronotype”),^10^ daytime napping frequency,^22^ snoring, insomnia symptoms,^9^ difficulty awakening, and daytime sleepiness^23^ (phenotype definitions and GWAS procedures are provided in Data S1 and Table S2). We selected all available sleep traits so as to provide an unbiased survey of the relationship between sleep health and migraine. The question used to define self‐reported insomnia symptoms in UKB has been shown to be sensitive and specific for clinically diagnosed insomnia disorder in an independent sample.^24^ Although daytime sleepiness is generally investigated as an outcome, we included it as an exposure here because the genetic architecture of daytime sleepiness suggests that the trait may partly reflect sleep fragmentation.^6^, ^23^ Genetic variants that associate with sleep traits in these GWAS also strongly associate with corresponding objective measures of sleep.^21^

#### Data sources: migraine GWAS

We obtained genetic associations with migraine from the largest available meta‐analysis of genome‐wide association studies (GWAS) of migraine conducted by the International Headache Genetics Consortium (IHGC).^8^ This study comprised 59,674 cases and 316,078 controls from 22 GWA studies (including 23andMe), conducted using data from six tertiary headache clinics (*n* = 20,395) and 27 population‐based cohorts (*n* = 355,357). Characteristics of each of the contributing cohorts have been previously described.^8^ Migraine cases were defined using a range of different approaches across the cohorts including self‐report, questionnaires assessing diagnostic criteria, and diagnosis by a trained clinician interviewer. All participants had genetically verified European ancestry.

### Genetic correlation analyses

We calculated genome‐wide genetic correlations (rg) using cross‐trait LD Score regression with precomputed LD scores^19^, ^25^ (Data S1). A positive genetic correlation differing from 0 implies that genetic variants increasing risk for one trait tend to also increase risk for the other trait.

### Mendelian randomization analyses

**Figure 1:**
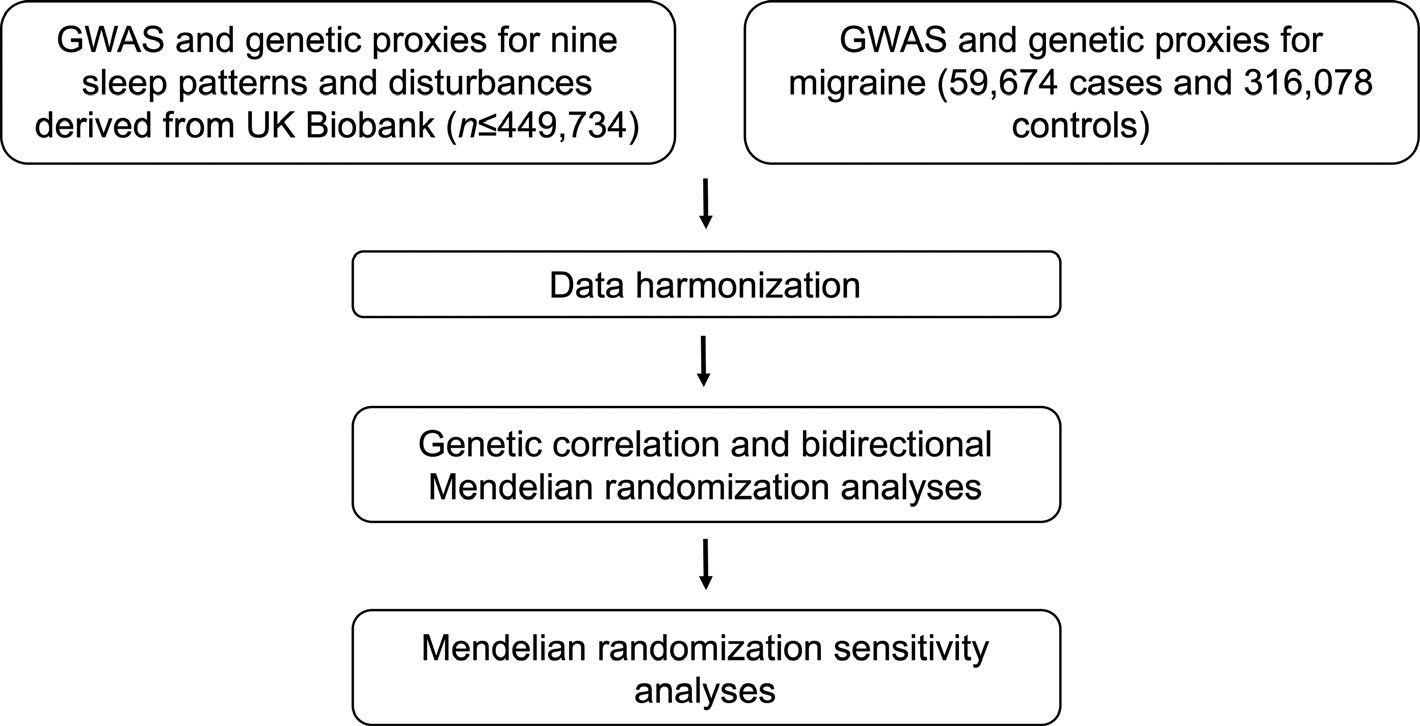
Mendelian randomization analysis pipeline. GWAS, genome‐wide association study; IHGC, international headache genetics consortium; UKB, UK Biobank

The design of our MR analysis is shown in Figure 1, with details of data harmonization provided in the Data S1. The primary MR method was random‐effects inverse‐variance weighted (IVW) regression,^26^ with sleep and migraine alternately used as exposure or outcome. For ordinal phenotypes (Table S1), a one‐unit increase in the genetic instrument corresponds to a unit increase in the ordinal scale. For dichotomous phenotypes, a one‐unit increase in the genetic instrument reflects a doubling in the odds of the exposure trait.^27^

### Sensitivity analyses

MR provides strong evidence for causality under the following assumptions^14^: (1) the genetic instrument is strongly associated with the exposure, (2) the genetic instrument is not associated with confounders, and (3) the genetic instrument only affects the outcome through its effect on the exposure (i.e., no horizontal pleiotropy).^14^ As the second MR assumption is generally satisfied by the use of randomly allocated alleles as instrumental variables and by control for population stratification in GWAS, we focused on approaches to address assumptions 1 and 3. Broadly, to address assumption 1 we performed sensitivity analyses using stronger genetic instruments for insomnia. To assess assumption 3, we used four models robust to various forms of pleiotropy, and tested for pleiotropy between the exposures and other sleep traits, and between the exposures and psychiatric comorbidities (depression and anxiety symptoms). Technical details regarding these sensitivity analyses are provided in the Data S1.

### Hypothesis testing and statistical software

The Bonferonni‐adjusted threshold for MR analyses accounted for 14 forward and reverse MR tests (without double‐counting short and long sleep duration, which are highly correlated with sleep duration measured continuously^12^), yielding an alpha threshold of 0.05/14 = 0.0036. The corrected alpha threshold in genetic correlation analyses was 0.05/7 = 0.007. *P* values less than these corrected alpha thresholds were considered to represent significant evidence for causal effects, and *P* < 0.05 was considered to represent nominal evidence for a causal effect. Analyses were performed using the LDSC software,^19^, ^25^ R version 3.5.0 and the TwoSampleMR^28^ package, and the GSMR^29^ software.^30^

### Standard protocol approvals, registrations, and patient consents

All UKB participants provided written informed consent, and all data used in this study were deidentified. Sleep GWAS data are available at the Sleep Disorder Knowledge portal (see data links). The IGHC migraine GWAS summary statistics including data from 23andMe were provided under a Data Transfer Agreement by 23andMe.

## Results

### Migraine shares genetic determinants with multiple sleep patterns and disturbances

As sleep disturbances are comorbid with migraine and are also heritable, we first tested if the traits have shared genetic influences using cross‐trait LD score regression.^25^ Migraine was genetically correlated with seven out of nine sleep patterns or disturbances after Bonferonni correction *P* < 0.007; Table 1). Insomnia symptoms had the strongest and most significant evidence for a shared genetic basis with migraine (rg [95% CI] 0.29 [0.25–0.33], *P* = 1.87 × 10^−32^), with weaker correlations between migraine and short sleep duration (0.18 [0.12–0.24], *P* = 1.69 × 10^−9^), difficulty awakening (0.11 [0.05–0.17], *P* = 2.02 × 10^−5^), and daytime napping (0.11 [0.05–0.17], *P* = 1.31 × 10^−5^). There was no evidence for a genetic correlation between migraine and morning diurnal preference (−0.03 [−0.07–0.01], *P* = 0.24) or snoring (0.01 [−0.05–0.07], *P* = 0.84).

**Table 1:** Genetic correlations between migraine and sleep traits

Sleep trait^1^	Genetic correlation with migraine (SE)	*P* value
Morning diurnal preference	−0.03 (0.02)	0.24
Difficulty awakening	0.11 (0.03)	2.02 × 10^−5*^
Insomnia symptoms	0.29 (0.02)	1.87 × 10^−32*^
Long sleep duration (≥9h)	0.12 (0.04)	7.60 × 10^−4*^
Short sleep duration (<7h)	0.18 (0.03)	1.69 × 10^−9*^
Sleep duration (hours)	−0.08 (0.03)	1.56 × 10^−3*^
Napping	0.11 (0.03)	1.31 × 10^−5*^
Daytime sleepiness	0.09 (0.03)	1.21 × 10^−4*^
Snoring	0.01 (0.03)	0.84

GWAS, genome‐wide association study; SE, standard error.

^1^ The LDSC intercept ranged from 1.02 (daytime sleepiness) to 1.06 (morning diurnal preference), consistent with the absence of uncontrolled confounding. Z scores for heritability were all greater than 4^19^, supporting the validity of genetic correlation analyses.

* *P* less than Bonferonni‐corrected threshold of 0.05/7 = 0.007.

### Mendelian randomization analyses support causal effects of difficulty awakening and insomnia symptoms on migraine

**Figure 2:**
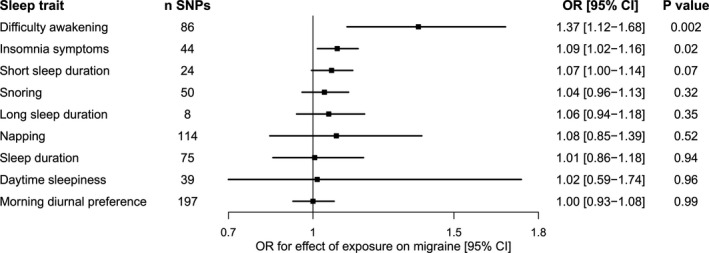
Forest plot of two‐sample Mendelian randomization estimates for effects of sleep phenotypes on risk of migraine (59,674 cases and 316,078 controls). Estimates were obtained using the random‐effects inverse‐variance weighted method. CI, confidence interval

To investigate whether any of the sleep traits causally influence migraine susceptibility, we performed two‐sample MR analyses using established genetic signals to proxy each of the sleep exposures (Fig. 2; Table S1). There was evidence for a significant effect of difficulty awakening on migraine (OR [95% CI] 1.37 [1.12–1.68], *P* = 0.002). There was also nominal evidence for an effect of liability to insomnia symptoms on migraine (1.09 [1.02–1.16], *P* = 0.015). Removing weakly correlated SNPs (using a stricter clumping threshold of *r* ^2^ < 0.001 vs. *r* ^2^ < 0.01) yielded nearly identical effect estimates for insomnia (36 SNPs; 1.09 [1.01–1.17], *P* = 0.019) and for difficulty awakening (71 SNPs; 1.37 [1.10–1.71], *P* = 0.006). MR estimates were null for the effect of all other sleep traits on migraine susceptibility (Fig. 2).

### Mendelian randomization estimates are robust in sensitivity analyses

We first tested whether results were consistent when using a genetic instrument for insomnia symptoms developed from a meta‐analysis of the UK Biobank and 23andme studies (*n* = 1.3 million^24^). Using this 195‐SNP genetic instrument, we found a slightly stronger and more significant estimate for a causal effect of liability to insomnia symptoms on migraine (1.14 [1.11–1.16], *P* = 7.64 × 10^−24^).

We next tested whether MR results were robust to sensitivity analyses assessing the validity of the assumption of no horizontal pleiotropy. The MR estimates were largely consistent in four model‐based sensitivity analyses for pleiotropy (Fig. S1; Table S6). Leave‐one‐out plots revealed that the rs113851554 variant in *MEIS1* flipped the Egger regression effect estimate for insomnia on migraine (Fig. S2). In analyses without this variant, the Egger effect estimate was directionally concordant with the IVW estimate but had wide confidence intervals (Fig. S1). No outliers were detected in any other leave‐one‐out analyses (Figs. S3–S5).

We next performed sensitivity analyses to determine whether the MR estimates were biased by pleiotropy with other sleep traits or with MDD. There was no evidence for a causal effect of liability to restless legs syndrome (RLS) on migraine susceptibility, suggesting that effects of insomnia symptoms on migraine are not driven by pleiotropic effects of the variants on RLS (1.03 [0.99–1.07], *P* = 0.10). The effects of insomnia symptoms and difficulty awakening on migraine were consistent when excluding variants associated at genome‐wide significance with other sleep traits, and in multivariable MR modeling pleiotropic effects on both exposures (Fig. S1). The MR estimates for the effect of insomnia symptoms on migraine were partly attenuated but remained significant in multivariable MR when adjusting for genetic associations with MDD or anxious symptoms (Fig. S1).

### Mendelian randomization does not support causal effects of migraine on sleep patterns and disturbances

**Figure 3:**
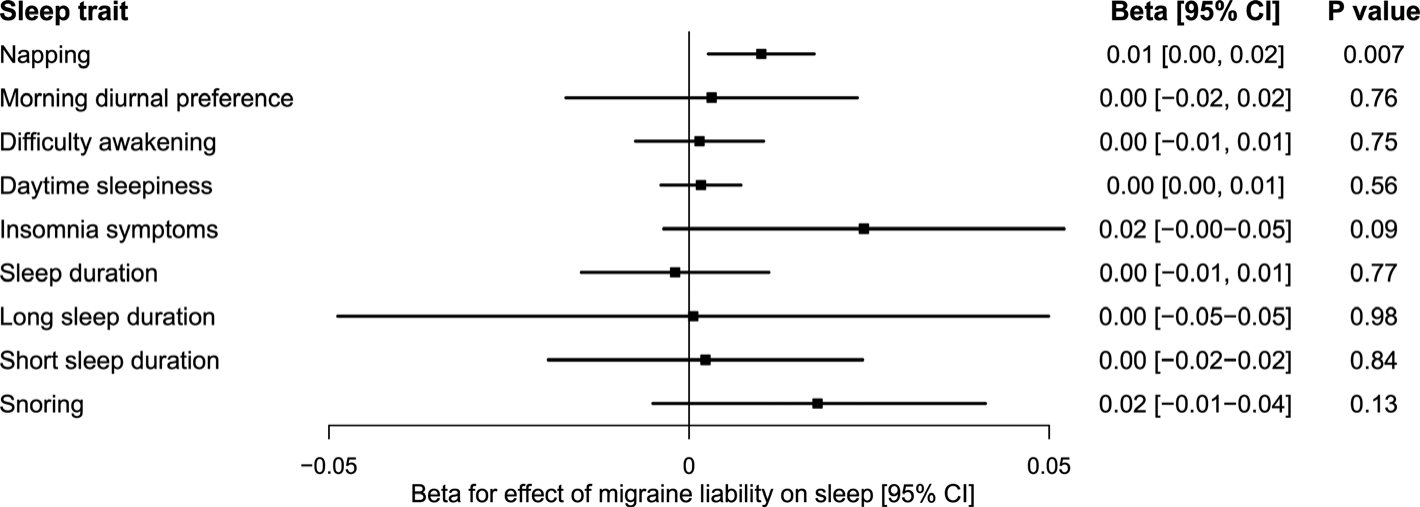
Forest plot of two‐sample Mendelian randomization estimates for effect of genetic liability of migraine on sleep traits. Thirty‐five single nucelotide polymorphisms were used as genetic proxies for migraine liability. Estimates were obtained using the random‐effects inverse‐variance weighted method. MR estimates for binary outcomes (insomnia symptoms, long sleep duration, short sleep duration, and snoring) are reported on the log‐odds scale. CI, confidence interval

We next assessed whether genetic liability to migraine impacted habitual sleep patterns and disturbances. Genetically predicted liability to migraine did not significantly influence any sleep disturbances, with confidence intervals excluding large effects (Fig. 3; Table S8). There was suggestive evidence for a weak effect of migraine liability on increased napping (0.01 unit increase in napping frequency [0.003, 0.017], *P* = 0.007), with consistent estimates across sensitivity analyses (Table S9).

## Discussion

We leveraged genetic methods to investigate comorbidity and causality between migraine and sleep disturbances. We found evidence for shared genetic influences between multiple sleep traits and migraine, as well as potential causal effects of insomnia symptoms and difficulty awakening on migraine. These effects were robust in sensitivity analyses for horizontal pleiotropy and there was no evidence for strong effects in the reverse direction.

We found evidence f shared genetic influences between several sleep traits and migraine, with the strongest genetic correlation found with insomnia symptoms (rg = 0.29). With the exception of a previously reported genetic correlation of migraine with MDD of 0.32, the magnitude of genetic overlap between insomnia and migraine was greater than that reported for most other common disease traits in the UK Biobank^18^ and in previous studies,^16^, ^31^, ^32^ suggesting more shared underlying biology between migraine and insomnia than migraine and other cardiometabolic, neuropsychiatric and immune phenotypes. Weaker but highly significant correlations of migraine were seen with other sleep duration and quality traits, confirming that the highly pleiotropic migraine genetic loci also influence sleep traits. As the sample size for migraine GWAS grows, future cross‐phenotype analyses may identify specific loci underlying these genetic correlations. Although prior work has demonstrated that *rare* mutations in the casein kinase (CK Iδ) gene may simultaneously cause familial migraine and advanced sleep phase syndrome,^33^ our work showed no evidence for an overall shared genetic basis for migraine and morning diurnal preference. This suggests that genetic variation in circadian rhythms may not generally have an important effect on migraine etiology, but certain circadian genes (e.g., CK Iδ) may have pleiotropic roles in migraine via pathways unrelated to their circadian effects.^33^

Mendelian randomization analyses suggested a causal effect of insomnia symptoms on migraine, adding support to findings from prospective epidemiologic studies.^7^ This estimate was consistent across sensitivity analyses, and was stronger in a secondary analysis using a larger number of insomnia SNPs from a meta‐analysis of UK Biobank and 23andMe. These variants were only used in sensitivity analyses because sample overlap of the insomnia symptoms GWAS (288,557 insomnia cases and 655,920 controls from 23andMe)^24^ with the migraine GWAS (30,465 migraine cases and 143,147 controls from 23andMe)^8^ may bias effect estimates away from the null. However, this bias is unlikely to be large given that the degree of case overlap is not large (up to 30,465 migraine GWAS cases included in the insomnia GWAS of *n* = 1,331,010; 3%) and that the genetic instrument for insomnia is strong (*F‐*statistic > 10).^34^ Given the nominal statistical evidence for this finding, additional replication in independent samples with well‐defined and validated diagnostic criteria for insomnia will strengthen confidence in this effect. Nevertheless, the evidence from this study supports findings from longitudinal epidemiologic studies of insomnia and migraine (reviewed by Uhlig et al.).^7^ One of the largest studies to date (26,197 participants from the HUNT study) reported that individuals with insomnia at baseline had a relative risk of 1.40 (95% CI 1.0–1.9; *P* = 0.02) for migraine after 11 years of follow up.^35^ Our results are also consistent with evidence from a clinical trial of cognitive behavioral therapy for insomnia in patients with migraine, in which treatment of insomnia reduced migraine frequency.^36^ Although insomnia symptoms are genetically correlated with short sleep duration,^9^, ^12^ there was no significant effect of genetically proxied self‐reported short sleep duration on migraine. This is in contrast to prior MR analyses which found concordant effects of insomnia and short sleep duration on coronary artery disease risk,^9^, ^37^ suggesting that the short sleep component of insomnia may be less relevant to the etiology of migraine. Rather, other features of insomnia such as hyperarousal may play more prominent roles in the etiology of migraine.^38^

Relative to insomnia, less is known about the phenomenon of difficulty awakening, which in some settings is referred to as sleep inertia.^39^, ^40^ Difficulty awakening is inversely genetically correlated^24^ with morning diurnal preference (rg = −0.78) and with insomnia symptoms (rg = 0.23) and may therefore reflect a combination of circadian misalignment and interrupted sleep.^24^, ^39^ However, we did not find evidence for a causal effect of morning diurnal preference on migraine. This suggests that the effect of difficulty awakening on migraine may be driven by disturbances to sleep quality rather than through circadian mechanisms. Difficulty awakening^40^ may also be a consequence of psychiatric comorbidities, and prior work has highlighted genetic correlations between sleep and psychiatric comorbidities,^9^, ^12^ and between migraine and psychiatric disease.^31^ This motivated multivariable MR analyses adjusting MR estimates for potential pleiotropy with MDD and with anxious symptoms. We found partial attenuation of the MR estimates for both difficulty awakening and insomnia symptoms on migraine when adjusting for MDD, however the adjusted MR estimate remained significant. This finding is consistent with prior epidemiologic analyses that have shown that sleep disturbances influence migraine risk independently of MDD and anxiety.^41^ This suggests that sleep disturbances directly influence migraine risk independently of psychiatric comorbidities and therefore warrant intervention in their own right.

There was minimal evidence for an effect of migraine on any of the sleep patterns or disturbances. While longitudinal epidemiologic studies lasting up to 11 years have suggested potential effects of migraine on insomnia risk,^7^, ^35^ our results are in line with microlongitudinal studies that have not shown effects of migraine headaches on next‐day sleep.^42^ We did, however, identify a small effect of migraine liability on increased napping frequency. The use of naps as an acute abortive treatment for migraine^2^ may be one possible mechanism mediating this effect. The generally null effects of migraine on habitual sleep patterns do not exclude an acute effect of a migraine episode on sleep. An analogy may be drawn to the relationship of caffeine with sleep, where MR analyses have not shown causal effects of caffeine on sleep patterns,^43^ suggesting discordance between effects of short and long‐term caffeine consumption. Similarly, while a migraine headache may acutely interrupt sleep, we did not find strong evidence for effects of migraine liability on sustained sleep patterns.

There are several potential pathways by which sleep quality or insomnia symptoms may influence migraine susceptibility. Cortical excitability, a potential mechanism of migraine pathophysiology,^44^ may be increased by insomnia.^45^ Sleep disturbances^2^, ^46^ may also reduce pain thresholds^47^ and cause dysfunction of the glymphatic system, resulting in accumulation of nociceptive CNS waste.^4^, ^41^ Finally, difficulty awakening may reflect slow clearance of CNS adenosine,^39^ with the consequent increases in adenosine increasing the likelihood of headache onset.^48^ Additional work is necessary to determine which of these pathways, if any, are relevant to the effect of sleep disturbances on migraine.

We acknowledge limitations to this work. First, although we incorporated sensitivity analyses for horizontal pleiotropy, we cannot fully exclude the influence of this potential bias. Second, MR power calculators are not currently designed for ordinal or binary exposures, so we focused on interpretation of the confidence intervals to determine whether the bounds contained clinically relevant effects. Third, single, self‐reported questions are less reliable for phenotyping than validated scales or physician‐diagnosed insomnia, which were unavailable in UKB. Fourth, the known common variant contributions to migraine primarily reflect the genetic architecture of migraine without aura (MO), which is the most prevalent form of migraine.^8^ Our findings may therefore have greater relevance to the pain component of migraine, which is more prominent in MO.^49^ This limitation may be addressed in future analyses as genetic data on migraine with aura become more robust. Finally, the selection of relatively healthy individuals into UKB may limit generalizability to less healthy populations and to populations of non‐European ancestry.

## Conclusion

The genetic determinants of sleep and migraine are partly overlapping. Sleep disturbances may causally influence migraine etiology, and are promising targets for the treatment of migraine.

## Author Contributions

ID and RS conceived and designed the study with input from all coauthors. RS and DC provided the data. ID and YG analyzed the data. ID drafted the initial manuscript. RS, AV, YG, and DC provided critical feedback to the manuscript and approved the final version. ID and RS are the guarantors. The corresponding authors attest that all listed authors meet authorship criteria and that no others meeting the criteria have been omitted.

## International Headache Genetics Consortium Members

Padhraig Gormley^31−34^, Verneri Anttila^32,33,35^, Bendik S. Winsvold^36−38^, Priit Palta^39^, Tonu Esko^32,40,41^, Tune H. Pers^32,41−43^, Kai‐How Farh^32,35,44^, Ester Cuenca‐Leon^31−33,45^, Mikko Muona^39,46−48^, Nicholas A. Furlotte^30^, Tobias Kurth^49,9^, Andres Ingason^10^, George McMahon^50^, Lannie Ligthart^51^, Gisela M. Terwindt^52^, Mikko Kallela^53^, Tobias M. Freilinger^54,55^, Caroline Ran^56^, Scott G. Gordon^22^, Anine H. Stam^52^, Stacy Steinberg^10^, Guntram Borck^57^, Markku Koiranen^58^, Lydia Quaye^59^, Hieab H. H. Adams^6,61^, Terho Lehtimäki^62^, Antti‐Pekka Sarin^39^, Juho Wedenoja^63^, David A. Hinds^30^, Julie E. Buring^9,64^, Markus Schürks^65^, Paul M. Ridker^9,64^, Maria Gudlaug Hrafnsdottir^66^, Hreinn Stefansson^10^, Susan M. Ring^50^, Jouke‐Jan Hottenga^51^, Brenda W. J. H. Penninx^67^, Markus Färkkilä^53^, Ville Artto^53^, Mari Kaunisto^39^, Salli Vepsäläinen^53^, Rainer Malik^55^, Andrew C. Heath^68^, Pamela A. F. Madden^68^, Nicholas G. Martin^22^, Grant W. Montgomery^8^, Mitja I. Kurki^31−33,39,69^, Mart Kals^40^, Reedik Mägi^40^, Kalle Pärn^40^, Eija Hämäläinen^39^, Hailiang Huang^32,33,35^, Andrea E. Byrnes^32,33,35^, Lude Franke^70^, Jie Huang^34^, Evie Stergiakouli^50^, Phil H. Lee^31−33^, Cynthia Sandor^71^, Caleb Webber^71^, Zameel Cader^72,73^, Bertram Muller‐Myhsok^74,75^, Stefan Schreiber^76^, Thomas Meitinger^77,78^, Johan G. Eriksson^79,8^, Veikko Salomaa^80^, Kauko Heikkilä^81^, Elizabeth Loehrer^60,82^, Andre G. Uitterlinden^83^, Albert Hofman^60^, Cornelia M. van Duijn^60^, Lynn Cherkas^59^, Linda M. Pedersen^36^, Audun Stubhaug^84,85^, Christopher S. Nielsen^84,86^, Minna Männikkö^58^, Evelin Mihailov^40^, Lili Milani^40^, Hartmut Göbel^87^, Ann‐Louise Esserlind^88^, Anne Francke Christensen^88^, Thomas Folkmann Hansen^89^, Thomas Werge^90,91,7^, Jaakko Kaprio^39,63,92^, Arpo J. Aromaa^80^, Olli Raitakari^93,94^, M. Arfan Ikram^60,61,95^, Tim Spector^59^, Marjo‐Riitta Järvelin^58,96−98^, Andres Metspalu^40^, Christian Kubisch^99^, David P. Strachan^100^, Michel D. Ferrari^52^, Andrea C. Belin^56^, Martin Dichgans^55,75^, Maija Wessman^39,46^, Arn M. J. M. van den Maagdenberg^52,101^, John‐Anker Zwart^36−38^, Dorret I. Boomsma^51^, George Davey Smith^50^, Kari Stefansson^10,102^, Nicholas Eriksson^30^, Mark J. Daly^32,33,35^, Benjamin M. Neale^32,33,35^, Jes Olesen^88^, Daniel I. Chasman^9^, Dale R. Nyholt^1^, Aarno Palotie^31−35,103^.

## International Headache Genetics Consortium Affiliations

^1^School of Biomedical Sciences, Faculty of Health, and Institute of Health and Biomedical Innovation, Queensland University of Technology, Brisbane, Queensland, Australia. ^2^Department of Epidemiology and Cancer Control, St. Jude Children’s Research Hospital, Memphis, Tennessee 38105, USA. ^3^23andMe, Inc., 899 W. Evelyn Avenue, Mountain View, California 94041, USA. ^4^School of Pharmacy and Biomedical Sciences, University of Central Lancashire, Preston PR1 2HE, United Kingdom. ^5^Department of Obstetrics and Gynecology, Niigata University Graduate School of Medical and Dental Sciences, Niigata 950‐2181, Japan. ^6^Department of Biomedicine ‐ Human Genetics, Aarhus University, DK‐8000 Aarhus, Denmark. ^7^iPSYCH, The Lundbeck Foundation Initiative for Integrative Psychiatric Research, DK‐2100 Copenhagen, Denmark. ^8^Institute for Molecular Bioscience, The University of Queensland, Brisbane, Queensland 4072, Australia. ^9^Divisions of Preventive Medicine, Department of Medicine, Brigham and Women’s Hospital, Harvard Medical School, Boston, MA, USA. ^10^deCODE Genetics/Amgen, 101 Reykjavik, Iceland. ^11^Department of Biostatistics, University of Liverpool, Liverpool L69 3GL, UK. ^12^Wellcome Trust Centre for Human Genetics, University of Oxford, Oxford OX3 7BN, UK.^13^KULeuven, Department of Development and Regeneration, Organ systems, 3000 Leuven, Belgium. ^14^Department of Obstetrics and Gynaecology, Leuven University Fertility Centre, University Hospital Leuven, 3000 Leuven, Belgium. ^15^Harvard T.H. Chan School of Public Health, Boston, Massachusetts 02115, USA. ^16^Channing Division of Network Medicine, Department of Medicine, Brigham and Women’s Hospital and Harvard Medical School, Boston, Massachusetts 02115, USA. ^17^Division of Preventive Medicine, Brigham and Women’s Hospital, Boston, Massachusetts 02215, USA. ^18^Institute of Medicine and Public Health, Vanderbilt University Medical Center, Nashville, Tennessee 37203, USA. ^19^Vanderbilt Genetics Institute, Division of Epidemiology, Institute of Medicine and Public Health, Department of Medicine, Vanderbilt University Medical Center, Nashville, Tennessee 37203, USA. ^20^Cognitive Science Department, University of California, San Diego, La Jolla, California 92093, USA. ^21^Institute of Biological Psychiatry, Mental Health Centre Sct. Hans, Copenhagen University Hospital, DK‐2100 Copenhagen, Denmark. ^22^Department of Genetics and Computational Biology, QIMR Berghofer Medical Research Institute, Brisbane, Queensland 4006, Australia. ^23^Endometriosis CaRe Centre, Nuffield Dept of Obstetrics & Gynaecology, University of Oxford, John Radcliffe Hospital, Oxford OX3 9DU, UK. ^24^Center for Integrative Medical Sciences, RIKEN, Yokohama 230‐0045, Japan. ^25^Institute of Medical Sciences, The University of Tokyo, Tokyo 108‐8639, Japan. ^26^Department of Obstetrics and Gynecology, Landspitali University Hospital, 101 Reykjavik, Iceland. ^27^Faculty of Medicine, School of Health Sciences, University of Iceland, 101 Reykjavik, Iceland. ^28^Vanderbilt Genetics Institute, Vanderbilt Epidemiology Center, Institute of Medicine and Public Health, Department of Obstetrics and Gynecology, Vanderbilt University Medical Center, Nashville, Tennessee 37203, USA. ^29^Global Medical Affairs Fertility, Research and Development, Merck KGaA, Darmstadt, Germany. ^30^23andMe, Inc., 899 W. Evelyn Avenue, Mountain View, California 94041, USA. ^31^Psychiatric and Neurodevelopmental Genetics Unit, Massachusetts General Hospital and Harvard Medical School, Boston, Massachusetts, USA. ^32^Medical and Population Genetics Program, Broad Institute of MIT and Harvard, Cambridge, Massachusetts, USA. ^33^Stanley Center for Psychiatric Research, Broad Institute of MIT and Harvard, Cambridge, Massachusetts, USA. ^34^Wellcome Trust Sanger Institute, Wellcome Trust Genome Campus, Hinxton, UK. ^35^Analytic and Translational Genetics Unit, Massachusetts General Hospital and Harvard Medical School, Boston, Massachusetts, USA. ^36^FORMI, Oslo University Hospital, Oslo, Norway. ^37^Department of Neurology, Oslo University Hospital, Oslo, Norway. ^38^Institute of Clinical Medicine, University of Oslo, Oslo, Norway. ^39^Institute for Molecular Medicine Finland (FIMM), University of Helsinki, Helsinki, Finland. ^40^Estonian Genome Center, University of Tartu, Tartu, Estonia. ^41^Division of Endocrinology, Boston Children’s Hospital, Boston, Massachusetts, USA. ^42^Department of Epidemiology Research, Statens Serum Institut, Copenhagen, Denmark. ^43^Novo Nordisk Foundation Center for Basic Metabolic Research, University of Copenhagen, Copenhagen, Denmark. ^44^Illumina, San Diego, California, USA. ^45^Pediatric Neurology, Vall d’Hebron Research Institute, Barcelona, Spain. ^46^Folkhälsan Institute of Genetics, Helsinki, Finland. ^47^Neuroscience Center, University of Helsinki, Helsinki, Finland. ^48^Molecular Neurology Research Program, Research Programs Unit, University of Helsinki, Helsinki, Finland. ^49^Institute of Public Health, Charité–Universitätsmedizin Berlin, Berlin, Germany. ^50^Medical Research Council (MRC) Integrative Epidemiology Unit, University of Bristol, Bristol, UK. ^51^Department of Biological Psychology, Vrije Universiteit, Amsterdam, the Netherlands. ^52^Department of Neurology, Leiden University Medical Centre, Leiden, the Netherlands. ^53^Department of Neurology, Helsinki University Central Hospital, Helsinki, Finland. ^54^Department of Neurology and Epileptology, Hertie‐Institute for Clinical Brain Research, University of Tuebingen, Tuebingen, Germany. ^55^Institute for Stroke and Dementia Research, Klinikum der Universität München, Ludwig‐Maximilians‐Universität München, Munich, Germany. ^56^Department of Neuroscience, Karolinska Institutet, Stockholm, Sweden. ^57^Institute of Human Genetics, Ulm University, Ulm, Germany. ^58^Center for Life Course Epidemiology and Systems Medicine, University of Oulu, Oulu, Finland. ^59^Department of Twin Research and Genetic Epidemiology, King’s College London, London, UK. ^60^Department of Epidemiology, Erasmus University Medical Center, Rotterdam, the Netherlands. ^61^Department of Radiology, Erasmus University Medical Center, Rotterdam, the Netherlands. ^62^Department of Clinical Chemistry, Fimlab Laboratories, School of Medicine, University of Tampere, Tampere, Finland. ^63^Department of Public Health, University of Helsinki, Helsinki, Finland. ^64^Harvard Medical School, Boston, Massachusetts, USA. ^65^Department of Neurology, University Duisburg–Essen, Essen, Germany. ^66^Landspitali University Hospital, Reykjavik, Iceland. ^67^Department of Psychiatry, VU University Medical Centre, Amsterdam, the Netherlands. ^68^Department of Psychiatry, Washington University School of Medicine, St. Louis, Missouri, USA. ^69^Department of Neurosurgery, NeuroCenter, Kuopio University Hospital, Kuopio, Finland. ^70^Department of Genetics, University Medical Center Groningen, University of Groningen, Groningen, the Netherlands. ^71^MRC Functional Genomics Unit, Department of Physiology, Anatomy & Genetics, Oxford University, Oxford, UK. ^72^Nuffield Department of Clinical Neuroscience, University of Oxford, Oxford, UK. ^73^Oxford Headache Centre, John Radcliffe Hospital, Oxford, UK. ^74^Max Planck Institute of Psychiatry, Munich, Germany. ^75^Munich Cluster for Systems Neurology (SyNergy), Munich, Germany. ^76^Institute of Clinical Molecular Biology, Christian Albrechts University, Kiel, Germany. ^77^Institute of Human Genetics, Helmholtz Zentrum München, Neuherberg, Germany. ^78^Institute of Human Genetics, Technische Universität München, Munich, Germany. ^79^Department of General Practice and Primary Health Care, University of Helsinki and Helsinki University Hospital, Helsinki, Finland. ^80^National Institute for Health and Welfare, Helsinki, Finland. ^81^Institute of Clinical Medicine, University of Helsinki, Helsinki, Finland. ^82^Department of Environmental Health, Harvard T.H. Chan School of Public Health, Boston, Massachusetts, USA. ^83^Department of Internal Medicine, Erasmus University Medical Center, Rotterdam, the Netherlands. ^84^Department of Pain Management and Research, Oslo University Hospital, Oslo, Norway. ^85^Medical Faculty, University of Oslo, Oslo, Norway. ^86^Department of Ageing and Health, Norwegian Institute of Public Health, Oslo, Norway. ^87^Kiel Pain and Headache Center, Kiel, Germany. ^88^Danish Headache Center, Department of Neurology, Rigshospitalet, Glostrup Hospital, University of Copenhagen, Copenhagen, Denmark. ^89^Institute of Biological Psychiatry, Mental Health Center Sct. Hans, University of Copenhagen, Roskilde, Denmark. ^90^Institute of Biological Psychiatry, MHC Sct. Hans, Mental Health Services Copenhagen, Copenhagen, Denmark. ^91^Institute of Clinical Sciences, Faculty of Medicine and Health Sciences, University of Copenhagen, Copenhagen, Denmark. ^92^Department of Health, National Institute for Health and Welfare, Helsinki, Finland. ^93^Research Centre of Applied and Preventive Cardiovascular Medicine, University of Turku, Turku, Finland. ^94^Department of Clinical Physiology and Nuclear Medicine, Turku University Hospital, Turku, Finland. ^95^Department of Neurology, Erasmus University Medical Center, Rotterdam, the Netherlands. ^96^Department of Epidemiology and Biostatistics, MRC Health Protection Agency (HPE) Centre for Environment and Health, School of Public Health, Imperial College London, London, UK. ^97^Biocenter Oulu, University of Oulu, Oulu, Finland. ^98^Unit of Primary Care, Oulu University Hospital, Oulu, Finland. ^99^Institute of Human Genetics, University Medical Center Hamburg‐Eppendorf, Hamburg, Germany. ^100^Population Health Research Institute, St George’s, University of London, London, UK. ^101^Department of Human Genetics, Leiden University Medical Centre, Leiden, the Net herlands. ^102^Faculty of Medicine, University of Iceland, Reykjavik, Iceland. ^103^Department of Neurology, Massachusetts General Hospital, Boston, Massachusetts, USA.

## Conflicts of Interest

The authors declare no conflicts of interest.

## Supporting information


**Data S1.** Supplementary Methods.
**Table S1.** Summary of GWAS and genetic instruments used in MR analysis.
**Table S2.** UK Biobank questions answered by participants at the baseline visit to ascertain sleep outcomes.
**Table S3.** Variants used in genetic instruments for sleep exposures.
**Table S4.** Validation of approach to selecting genetic instrumental variables for insomnia symptoms by comparison with lead variants identified in the insomnia symptoms GWAS.
**Table S5.** Variants used in the IHGC migraine genetic instrument (59,674 cases and 316,078 controls).
**Table S6.** Mendelian randomization heterogeneity and pleiotropy test results for significant effects identified in inverse‐variance weighted analysis.
**Table S7.** Variants removed in GSMR HEIDI filtering.
**Table S8.** MR estimates for the effect of migraine liability on binary sleep exposures, reported as odds ratios.
**Table S9.** MR sensitivity analyses for the effect of migraine liability on napping.
**Figure S1.** Forest plot of two‐sample Mendelian randomization sensitivity analyses for the effect of difficulty awakening and liability to insomnia symptoms on risk of migraine (59,674 cases and 316,078 controls).
**Figure S2.** Leave‐one‐out plot for MR Egger effect of liability to insomnia symptoms on risk of migraine.
**Figure S3.** Leave‐one‐out plot for MR Egger effect of difficulty awakening on risk of migraine.
**Figure S4.** Leave‐one‐out MR estimates for the effect of difficulty awakening on risk of migraine.
**Figure S5.** Leave‐one‐out MR estimates for the effect of insomnia symptoms on risk of migraine.

## Data Availability

Sleep GWAS data are available on the Sleep Disorder Knowledge Portal: http://www.kp4cd.org/dataset_downloads/sleep. The IHGC migraine GWAS data are available upon request to 23andMe: https://research.23andme.com/dataset‐access/.
