# Supplementary material for: Habitual sleep disturbances and migraine: a Mendelian randomization study
Source: Ann Clin Transl Neurol. 2020 Oct 30;7(12):2370–80. doi: 10.1002/acn3.51228 (PMC7732254; doi:10.1002/acn3.51228)
Supplement: Supplementary file 1 — Data S1. Supplementary Methods. Table S1. Summary of GWAS and genetic instruments used in MR analysis. Table S2. UK Biobank questions answered by participants at the baseline visit to ascertain sleep outcomes. Table S3. Variants used in genetic instruments for sleep exposures. Table S4. Validation of approach to selecting genetic instrumental variables for insomnia symptoms by comparison with lead variants identified in the insomnia symptoms GWAS. Table S5. Variants used in the IHGC migraine genetic instrument (59,674 cases and 316,078 controls). Table S6. Mendelian randomization heterogeneity and pleiotropy test results for significant effects identified in inverse‐variance weighted analysis. Table S7. Variants removed in GSMR HEIDI filtering. Table S8. MR estimates for the effect of migraine liability on binary sleep exposures, reported as odds ratios. Table S9. MR sensitivity analyses for the effect of migraine liability on napping. Figure S1. Forest plot of two‐sample Mendelian randomization sensitivity analyses for the effect of difficulty awakening and liability to insomnia symptoms on risk of migraine (59,674 cases and 316,078 controls). Figure S2. Leave‐one‐out plot for MR Egger effect of liability to insomnia symptoms on risk of migraine. Figure S3. Leave‐one‐out plot for MR Egger effect of difficulty awakening on risk of migraine. Figure S4. Leave‐one‐out MR estimates for the effect of difficulty awakening on risk of migraine. Figure S5. Leave‐one‐out MR estimates for the effect of insomnia symptoms on risk of migraine. [file ACN3-7-2370-s001.docx]

**Supplement**

**Supplementary methods**

*Details pertaining to the exposures used for the sleep GWAS*

UK Biobank (UKB) is a population-based cohort study that enrolled over 500,000 UK participants aged 40-69 from 2006-2010^1^. Nine million individuals were invited to participate in the study, of whom 5.5% enrolled. Enrolled participants completed a baseline questionnaire and standardized in-person interview with a study nurse, and blood was collected for genotyping. Full details regarding the cohort, including genotyping, imputation, and quality control procedures, are described in detail in prior publications^1,2^.

Sleep exposures included nine self-reported sleep patterns and sleep disturbances reported by participants in UKB. The phenotypes used in the study are summarized in Supplementary Table 1, and the questions and answer choices for these phenotypes are summarized in Supplementary Table 2. Phenotypic and genetic correlations of the sleep phenotypes in UKB have been previously studied^3^, and show strong correlations between the following phenotypes: difficulty awakening and morning diurnal preference (rg=-0.78), insomnia symptoms and sleep duration (-0.47), insomnia symptoms and difficulty awakening (0.23), and daytime sleepiness and napping (0.65). GWAS for these phenotypes were performed using BOLT-LMM^4^ to include related individuals, and were adjusted for age, sex, the first ten principal components of ancestry, and genotyping array. A SNP minor allele frequency cutoff of 0.001 and imputation quality score cutoff of 0.80 was used for analysis.

*Details pertaining to the IHGC migraine GWAS*

A SNP minor allele frequency cutoff of 0.01, imputation quality score cutoff of 0.60, and I^2^ meta-analysis heterogeneity cutoff of 0.75 between component studies were imposed for analysis.

*Genetic correlations*

We calculated genome-wide genetic correlations (rg) using cross-trait LD Score regression with pre-computed LD scores (sum of linkage disequilibrium (LD) *r*^2^ measured to all other SNPs from individuals of European ancestry from the 1000 genomes project)^5,6^. We restricted analysis to variants available in the HapMap3 panel, and used the following quality control criteria to filter variants: strand ambiguous, MAF less than 0.01, and alleles not matching to the 1000 Genomes European sample^5,6^. LDSC estimates genetic correlation (ranging approximately from -1 to 1) between two traits from GWAS summary statistics based on the fact that 1) GWAS effect-size estimate for each SNP incorporates effects of all SNPs in LD with that SNP, 2) SNPs with high LD have higher test statistics than SNPs with low LD, and 3) a similar relationship is observed when single study test statistics are replaced with the product of z-scores from two studies of traits. A positive genetic correlation differing from 0 implies that genetic variants increasing liability for one trait tend to increase liability for the other trait. Notably, rg can be slightly greater than 1 for highly correlated phenotypes^7^.

*Mendelian randomization analyses*

To identify SNPs available in both the sleep exposure and migraine outcome datasets, we first extracted all genome-wide significant variants associated with each sleep phenotype. We then restricted this list to SNPs also available in the IHGC GWAS and harmonized the exposure and outcome datasets by matching alleles. When possible, strand-ambiguous SNPs were aligned on the basis of allele identity and frequency (using the ‘harmonise_data’ function in the TwoSampleMR package^8^). We clumped this dataset using a clumping distance of 10Mb and *r*^2^ threshold of 0.01 to generate an independent set of variants for each exposure (using linkage disequilibrium estimates from the 1,000 Genomes European reference panel integrated within the TwoSampleMR^8^ software). In sensitivity analyses, we used an *r*^2^ threshold of 0.001 to exclude the possibility that residual LD may explain any observed effects. The selected variants are provided in Supplementary Table 3. As an example of the efficiency of this approach, for insomnia, the 44 selected SNPs had a median *r*^2^=1 (standard deviation 0.08) to the corresponding lead GWAS SNPs reported by Lane et al., and we identified a new lead variant (rs2297787) (Supplementary Table 4). We repeated the process in reverse for analyses of migraine on sleep (Supplementary Table 5).

*Sensitivity analyses to address MR assumptions*

We satisfied the first MR assumption by using genetic variants exceeding genome-wide significance for association with the exposure in each respective GWAS. We calculated the mean F statistic^9^ for each genetic instrument and used a threshold of F > 10 to ensure adequate instrument strength (Supplementary Table 1). The mean *F* statistic was greater than 38 for all instruments, suggesting minimal influence of weak instrument bias (Supplementary Table 1). As a sensitivity analysis for instrument strength, we used a larger GWAS to generate a stronger genetic instrument for insomnia symptoms^3^ comprising 195 variants available in the IHGC dataset and clumped these SNPs using a 10Mb distance and *r*^2^=0.01.

We conducted multiple sensitivity analyses to test the MR assumption that the genetic instrument only affects the outcome through its effect on the exposure^10^ (no horizontal pleiotropy). When there was evidence for heterogeneity in effect estimates between the SNPs in the genetic instrument using Cochran’s Q, we estimated effects using the weighted median^11^, MR Egger^12^ (and conducted a statistical test of significance for the Egger intercept, which represents the burden of unbalanced horizontal pleiotropy), MR-PRESSO, and generalized summary-data-based Mendelian randomization with HEIDI filtering (GSMR). We used leave-one-out plots for IVW estimates to confirm that effects were not unduly influenced by outliers potentially representing pleiotropic pathways. MR Egger is particularly influenced by outliers^13,14^, so we also checked leave-one-out plots for outliers potentially biasing effect estimates from the Egger regression and re-estimated the Egger effect without inclusion of outliers.

Given the known genetic overlap between the sleep traits, and between sleep traits and poor mental health^2,15–17^, we conducted sensitivity analyses to verify that pleiotropy between sleep phenotypes and poor mental health did not bias causal effects. First, given prior epidemiologic associations of restless legs syndrome (RLS) with migraine^18^, and the strong genetic overlap of RLS with insomnia symptoms^2^, we tested whether liability to restless legs syndrome (RLS) associated with migraine. As the RLS GWAS^19^ included 23andMe, we conducted MR with migraine in UKB as the outcome to generate unbiased two-sample MR estimates. Second, we conducted analyses excluding genetic instruments associated with more than one sleep phenotype at genome-wide significance in UKB. This threshold was selected to balance removal of horizontally pleiotropic variants while minimizing the removal of variants associated with multiple phenotypes through vertical pleiotropy (e.g. insomnia symptoms causing daytime sleepiness). Third, we used multivariable MR^20^ to model potential pleiotropy between the sleep traits, and between the sleep traits and major depressive disorder (MDD) and anxiety symptoms (GWAS details in Supplementary Methods)^21,22^. Attenuation of the effect of the sleep exposure in these analyses may be consistent with either horizontal or vertical (e.g. insomnia symptoms influencing migraine through its effects on depression) pleiotropy through MDD. Results from these analyses should therefore be considered an overly conservative test for potential bias due to horizontal pleiotropy.

*Major depressive disorder GWAS*

The GWAS of MDD combined genetic data from the Psychiatric Genomics Consortium (PGC) and UKB. The PGC meta-analyzed 33 cohorts which have been previously described in detail^21^, and cases were identified through a ascertained by trained interviewers, clinician-administered checklists, and medical record review ^21^. The UKB analysis used a broad definition of MDD cases (n=113,769 cases and 208,811 controls), classifying participants as cases who answered in the affirmative to either of the following questions: “Have you ever seen a general practitioner (GP) for nerves, anxiety, tension or depression?” and “Have you ever seen a psychiatrist for nerves, anxiety, tension or depression?”^22^ The PGC and UKB GWAS were meta-analyzed (n=170,756 cases / 329,443 controls) and made available on the PGC website (<https://www.med.unc.edu/pgc/download-results/)>.

*Anxiety symptoms GWAS*

There are no large, publically available GWAS that have been performed for the outcome of clinically diagnosed anxiety. We therefore used genetic associations from a GWAS of anxiety *symptoms* which was conducted using data from the UKB (*n*= 251,982 cases / 192,422 controls)^23^. The phenotype was ascertained through the question “"Are you a worrier?" This question is part of an assessment for neuroticism and is not designed for the diagnosis of generalized anxiety disorder^24^.

**Supplementary references**

1. Bycroft C, Freeman C, Petkova D, et al. The UK Biobank resource with deep phenotyping and genomic data. Nature 2018;562(7726):203–209.

2. Lane JM, Jones SE, Dashti HS, et al. Biological and clinical insights from genetics of insomnia symptoms. Nat. Genet. 2019;51(3):387–393.

3. Jansen PR, Watanabe K, Stringer S, et al. Genome-wide analysis of insomnia in 1,331,010 individuals identifies new risk loci and functional pathways. Nat. Genet. 2019;51(3):394–403.

4. Loh P-R, Tucker G, Bulik-Sullivan BK, et al. Efficient Bayesian mixed-model analysis increases association power in large cohorts. Nat. Genet. 2015;47(3):284–290.

5. Bulik-Sullivan BK, Loh P-R, Finucane HK, et al. LD Score regression distinguishes confounding from polygenicity in genome-wide association studies. Nat. Genet. 2015;47(3):291–295.

6. Bulik-Sullivan B, Finucane HK, Anttila V, et al. An atlas of genetic correlations across human diseases and traits. Nat. Genet. 2015;47(11):1236–1241.

7. Baselmans BML, Jansen R, Ip HF, et al. Multivariate genome-wide analyses of the well-being spectrum. Nat. Genet. 2019;51(3):445–451.

8. Hemani G, Zheng J, Elsworth B, et al. The MR-Base platform supports systematic causal inference across the human phenome. Elife 2018;7:e34408.

9. Zhao J V., Schooling CM. Thyroid function and ischemic heart disease: A Mendelian randomization study. Sci. Rep. 2017;7(1):1–11.

10. Davies NM, Holmes M V., Davey Smith G. Reading Mendelian randomisation studies: A guide, glossary, and checklist for clinicians. BMJ 2018;362

11. Bowden J, Davey Smith G, Haycock PC, Burgess S. Consistent Estimation in Mendelian Randomization with Some Invalid Instruments Using a Weighted Median Estimator. Genet. Epidemiol. 2016;40(4):304–314.

12. Bowden J, Smith GD, Burgess S. Mendelian randomization with invalid instruments: Effect estimation and bias detection through Egger regression. Int. J. Epidemiol. 2015;44(2):512–525.

13. Corbin LJ, Richmond RC, Wade KH, et al. BMI as a modifiable risk factor for type 2 diabetes: Refining and understanding causal estimates using mendelian randomization. Diabetes 2016;65(10):3002–3007.

14. Daghlas I, Dashti HS, Lane J, et al. Sleep Duration and Myocardial Infarction. J. Am. Coll. Cardiol. 2019;74(10):1304–1314.

15. Dashti HS, Jones SE, Wood AR, et al. Genome-wide association study identifies genetic loci for self-reported habitual sleep duration supported by accelerometer-derived estimates. Nat. Commun. 2019;10(1):1100.

16. Jones SE, Lane JM, Wood AR, et al. Genome-wide association analyses of chronotype in 697,828 individuals provides insights into circadian rhythms. Nat. Commun. 2019;10(1):343.

17. Wang H, Lane JM, Jones SE, et al. Genome-wide association analysis of self-reported daytime sleepiness identifies 42 loci that suggest biological subtypes. Nat. Commun. 2019;10(1):3503.

18. Schürks M, Winter AC, Berger K, et al. Migraine and restless legs syndrome in women. Cephalalgia 2012;32(5):382–389.

19. Schormair B, Zhao C, Bell S, et al. Identification of novel risk loci for restless legs syndrome in genome-wide association studies in individuals of European ancestry: a meta-analysis. Lancet Neurol. 2017;16(11):898–907.

20. Burgess S, Thompson SG. Multivariable Mendelian randomization: The use of pleiotropic genetic variants to estimate causal effects. Am. J. Epidemiol. 2015;181(4):251–260.

21. Wray NR, Ripke S, Mattheisen M, et al. Genome-wide association analyses identify 44 risk variants and refine the genetic architecture of major depression. Nat. Genet. 2018;50(5):668–681.

22. Howard DM, Adams MJ, Shirali M, et al. Genome-wide association study of depression phenotypes in UK Biobank identifies variants in excitatory synaptic pathways. Nat. Commun. 2018;9(1):1–10.

23. Jiang L, Zheng Z, Qi T, et al. A resource-efficient tool for mixed model association analysis of large-scale data. Nat. Genet. 2019;51(12):1749–1755.

24. Luciano M, Hagenaars SP, Davies G, et al. Association analysis in over 329,000 individuals identifies 116 independent variants influencing neuroticism. Nat. Genet. 2018;50(1):6–11.

**Supplementary Table 1.** **Summary of GWAS and genetic instruments used in MR analysis.**

| **Phenotype**  **(n cases/controls)** | **Used in primary or secondary analysis?** | **GWAS Author**  **[PMID]** | **Variable type** | ***n* SNPs in analysis** | **Mean F-statistic of genetic instruments [Range]** |
| --- | --- | --- | --- | --- | --- |
| Migraine - IHGC  (59,674 / 316,078) | Primary | Gormley  [27322543] | Binary | 35 | 56.6  [30-217] |
| Continuous sleep duration (446,118) | Primary | Dashti  [30846698] | Ordinal | 75 | 41  [30-221] |
| Short sleep duration (106,192 / 305,742) | Primary | Dashti  [30846698] | Binary | 24 | 38  [30-77] |
| Long sleep duration  (34,184 / 305,742) | Primary | Dashti  [30846698] | Binary | 8 | 40  [30-53] |
| Napping (452,683) | Primary | Unpublished  [UK Biobank] | Ordinal | 115 | 46  [30-217] |
| Morning diurnal preference (449,734) | Primary | Jones  [30696823] | Ordinal | 197 | 45  [29-221] |
| Snoring  (421,466) | Primary | Unpublished  [UK Biobank] | Binary | 50 | 39  [39-75] |
| Daytime sleepiness (452,071) | Primary | Wang  [31409809] | Ordinal | 39 | 42  [31-117] |
| Insomnia symptoms^2^  (129,270 / 108,357) | Primary | Lane  [30804566] | Binary | 44 | 42  [29-182] |
| Insomnia symptoms  (397,959 / 933,051) | Secondary | Jansen  [30804565] | Binary | 195 | 42  [26-217] |
| Difficulty awakening  (451,872) | Primary | Unpublished  [UK Biobank] | Ordinal | 86 | 42  [29-128] |
| Restless legs syndrome  (45,896 / 382,638) | Secondary | Schormair [29029846] | Binary | 20 | 280  [25-1435] |

GWAS: genome-wide association study; MR: Mendelian randomization; RLS: restless legs syndrome

**^1^**Participants reporting ‘usual’ insomnia symptoms were compared to those reporting ‘never/rarely’ experiencing insomnia symptoms.

**^2^**Participants reporting ‘usual’ insomnia symptoms were compared to those reporting either ‘sometimes’ or ‘never/rarely’ experiencing insomnia symptoms.

**^3^**Not used as an exposure.

**Supplementary Table 2. UK Biobank questions answered by participants at the baseline visit to ascertain sleep outcomes**.

| **Sleep trait or disturbance** | **Question** | **Answers** |
| --- | --- | --- |
| Sleep duration | About how many hours sleep do you get in every 24 hours? (please include naps) | Sleep duration in hour increments |
| Morning diurnal preference | Do you consider yourself to be? | 1. Definitely a 'morning' person  2. More a 'morning' than 'evening' person  3. More an 'evening' than a 'morning' person  4. Definitely an 'evening' person |
| Napping | Do you have a nap during the day? | 1. Never/rarely  2. Sometimes  3. Usually |
| Snoring | Does your partner or a close relative or friend complain about your snoring? | 1. Yes  2. No |
| Insomnia symptoms^1^ | Do you have trouble falling asleep at night or do you wake up in the middle of the night? | 1. Never/rarely  2. Sometimes  3. Usually |
| Difficulty awakening | On an average day, how easy do you find Ease of awakening in the morning? | 1. Not at all easy  2. Not very easy  3. Fairly easy  4. Very easy |
| Daytime sleepiness | How likely are you to doze off or fall asleep during the daytime when you don't mean to? (e.g. when working, reading or driving | 1. Never/rarely  2. Sometimes  3. Often  4. All of the time |

**^1^**The UKB insomnia instrument uses ‘usually’ as cases and ‘never/rarely’ as controls. In contrast, the expanded UKB-23andMe insomnia instrument defined controls as those reporting ‘never/rarely’ or ‘sometimes.’

**Supplementary Table 3. Variants used in genetic instruments for sleep exposures.**

*Table provided at the end of the document.*

**Supplementary Table 4. Validation of approach to selecting genetic instrumental variables for insomnia symptoms by comparison with lead variants identified in the insomnia symptoms GWAS.**

| **Instrumental SNP** | **Chromosome:Position** | **GWAS lead variant** | **Pair-wise LD estimate (r^2^)** |
| --- | --- | --- | --- |
| rs11184946 | chr1:107185225 | rs11184946 | 1 |
| rs6664467 | chr1:151738403 | rs6664467 | 1 |
| rs4648629 | chr1:1695462 | rs4751 | 0.96 |
| rs12405761 | chr1:57850914 | rs12405761 | 1 |
| rs11804386 | chr1:87738947 | rs11804386 | 1 |
| rs2296580 | chr10:104241683 | rs2296580 | 1 |
| rs2297787 | chr10:104680137 | rs2296580 | 0.0008^1^ |
| rs79780963 | chr10:104952499 | rs11191595 | NA^2^ |
| rs10838708 | chr11:47441513 | rs10838708 | 1 |
| rs12424599 | chr12:109866615 | rs68094047 | 0.99 |
| rs324017 | chr12:57487814 | rs324017 | 1 |
| rs2956278 | chr12:84698234 | rs2956278 | 1 |
| rs1923770 | chr13:53786568 | rs1923770 | 1 |
| rs1031654 | chr13:54382035 | rs1031654 | 1 |
| rs4886140 | chr13:59833519 | rs4886140 | 1 |
| rs11635495 | chr15:67804682 | rs11635495 | 1 |
| rs4886860 | chr15:74340336 | rs4886860 | 1 |
| rs1544637 | chr16:51484837 | rs1544637 | 1 |
| rs3104778 | chr16:52633652 | rs3104778 | 1 |
| rs2062113 | chr16:59476179 | rs2062113 | 1 |
| rs17139246 | chr16:6106260 | rs17139246 | 1 |
| rs17669584 | chr17:28899614 | rs17669584 | 1 |
| rs9894577 | chr17:43223292 | rs11651809 | 0.52 |
| rs1942262 | chr18:52873317 | rs1942262 | 1 |
| rs11673344 | chr19:37684966 | rs11673344 | 1 |
| rs7556815 | chr2:114085785 | rs62158170 | 0.96 |
| rs4577309 | chr2:191288833 | rs4577309 | 1 |
| rs35881094 | chr2:58922921 | rs35881094 | 1 |
| rs113851554 | chr2:66750564 | rs113851554 | 1 |
| rs9845387 | chr3:116425935 | rs9845387 | 1 |
| rs4688760 | chr3:49980596 | rs4688760 | 1 |
| rs11097861 | chr4:105330133 | rs11097861 | 1 |
| rs1381352 | chr4:91290773 | rs1841625 | 0.99 |
| rs28061 | chr5:102543878 | rs28061 | 1 |
| rs1592757 | chr5:103889998 | rs1592757 | 1 |
| rs7711696 | chr5:135486536 | rs7711696 | 1 |
| rs1430205 | chr5:87678585 | rs1430205 | 1 |
| rs6932158 | chr6:101246010 | rs6932158 | 1 |
| rs314280 | chr6:105400837 | rs314280 | 1 |
| rs10947690 | chr6:37631768 | rs10947690 | 1 |
| rs4723649 | chr7:1071905 | rs3824081 | 0.73 |
| rs6593005 | chr7:52584625 | rs6593005 | 1 |
| rs17151854 | chr8:10236559 | rs17151854 | 1 |
| rs10156602 | chr9:96345328 | rs10156602 | 1 |

^1^The clumping approach employed in the present study identified an independent genetic variant in this locus.

^2^Variant not present in 1000 Genomes European reference panel.

**Supplementary Table 5.** Variants used in the IHGC migraine genetic instrument (59,674 cases and 316,078 controls). EA: effect allele; IHGC: international headache genetics consortium; OA: other allele; EAF: effect allele frequency; SE: standard error.

| **SNP** | **EA** | **OA** | **Beta - IHGC** | **SE - IHGC** | **EAF** |
| --- | --- | --- | --- | --- | --- |
| rs10155855 | T | A | 0.081 | 0.014 | 0.054 |
| rs10166942 | T | C | 0.096 | 0.009 | 0.805 |
| rs10218452 | G | A | 0.108 | 0.008 | 0.223 |
| rs10456100 | T | C | 0.055 | 0.008 | 0.282 |
| rs10895275 | A | T | 0.041 | 0.007 | 0.327 |
| rs11031122 | C | T | 0.044 | 0.008 | 0.239 |
| rs11172113 | T | C | 0.104 | 0.007 | 0.580 |
| rs11624776 | A | C | 0.044 | 0.008 | 0.688 |
| rs72836764 | G | A | 0.088 | 0.014 | 0.930 |
| rs1268083 | T | C | 0.040 | 0.007 | 0.521 |
| rs13078967 | A | C | 0.144 | 0.024 | 0.972 |
| rs138556413 | C | T | 0.127 | 0.023 | 0.966 |
| rs144017103 | C | T | 0.187 | 0.034 | 0.978 |
| rs1572668 | G | A | 0.039 | 0.007 | 0.478 |
| rs17857135 | C | T | 0.062 | 0.010 | 0.167 |
| rs77410344 | C | T | 0.090 | 0.011 | 0.105 |
| rs1925950 | G | A | 0.069 | 0.007 | 0.351 |
| rs2078371 | C | T | 0.106 | 0.010 | 0.116 |
| rs2223089 | G | C | 0.071 | 0.013 | 0.918 |
| rs2506142 | G | A | 0.054 | 0.009 | 0.172 |
| rs28455731 | T | G | 0.054 | 0.009 | 0.161 |
| rs4081947 | G | A | 0.041 | 0.007 | 0.338 |
| rs4814864 | C | G | 0.070 | 0.008 | 0.257 |
| rs4839827 | C | T | 0.044 | 0.007 | 0.475 |
| rs4910165 | G | C | 0.056 | 0.007 | 0.669 |
| rs561561 | A | T | 0.062 | 0.011 | 0.877 |
| rs566529 | G | T | 0.058 | 0.010 | 0.853 |
| rs6478241 | A | G | 0.051 | 0.007 | 0.364 |
| rs6693567 | C | T | 0.045 | 0.008 | 0.272 |
| rs6791480 | T | C | 0.043 | 0.007 | 0.310 |
| rs75213074 | C | T | 0.111 | 0.019 | 0.967 |
| rs7544256 | A | G | 0.041 | 0.007 | 0.643 |
| rs75473620 | A | T | 0.112 | 0.019 | 0.960 |
| rs7684253 | T | C | 0.041 | 0.007 | 0.550 |
| rs9349379 | A | G | 0.069 | 0.007 | 0.590 |

**Supplementary Table 6**. Mendelian randomization heterogeneity and pleiotropy test results for significant effects identified in inverse-variance weighted analysis.

| **Exposure** | **Outcome** | **Cochran’s Q**  **P value** | **Egger intercept test P value** |
| --- | --- | --- | --- |
| Difficulty awakening | Migraine | 1.94e-06 | 0.76 |
| Insomnia symptoms | Migraine | 4.91e-09 | 0.34 |
| Migraine | Napping | 7.72e-04 | 0.73 |

**Supplementary Table 7.** Variants removed in GSMR HEIDI filtering.

| **Phenotype** | **Filtered variants** |
| --- | --- |
| Insomnia symptoms | rs113851554, rs7556815, rs324017, rs75651796, rs11673344, rs11635495, rs4723649, rs17151854, rs1544637 |
| Difficulty awakening | rs1421085, rs4916907, rs3760185, rs11075924, rs620598, rs8000625, rs2147324, rs62263580, rs34757401, rs6697589 |

**Supplementary Table 8. MR estimates for the effect of migraine liability on binary sleep exposures, reported as odds ratios.** CI: confidence interval.

| **Outcome** | **Beta [95% CI]** | ***P* value** |
| --- | --- | --- |
| Insomnia symptoms | 1.02 [1.00-1.05] | 0.09 |
| Long sleep duration | 1.00 [0.95-1.05] | 0.98 |
| Short sleep duration | 1.00 [0.98-1.02] | 0.84 |
| Snoring | 1.02 [1.00-1.04] | 0.13 |

**Supplementary Table 9. MR sensitivity analyses for the effect of migraine liability on napping.** CI: confidence interval; IVW: inverse-variance weighted; MR: Mendelian randomization; WM: weighted median

| **MR analysis** | **Beta [95% CI]** | ***P* value** |
| --- | --- | --- |
| IVW | 0.01 [0.00-0.02] | 0.007 |
| Egger | 0.02 [-0.01-0.03] | 0.21 |
| WM | 0.01 [0.00-0.02] | 0.03 |
| MR-PRESSO | No outliers detected | |

**
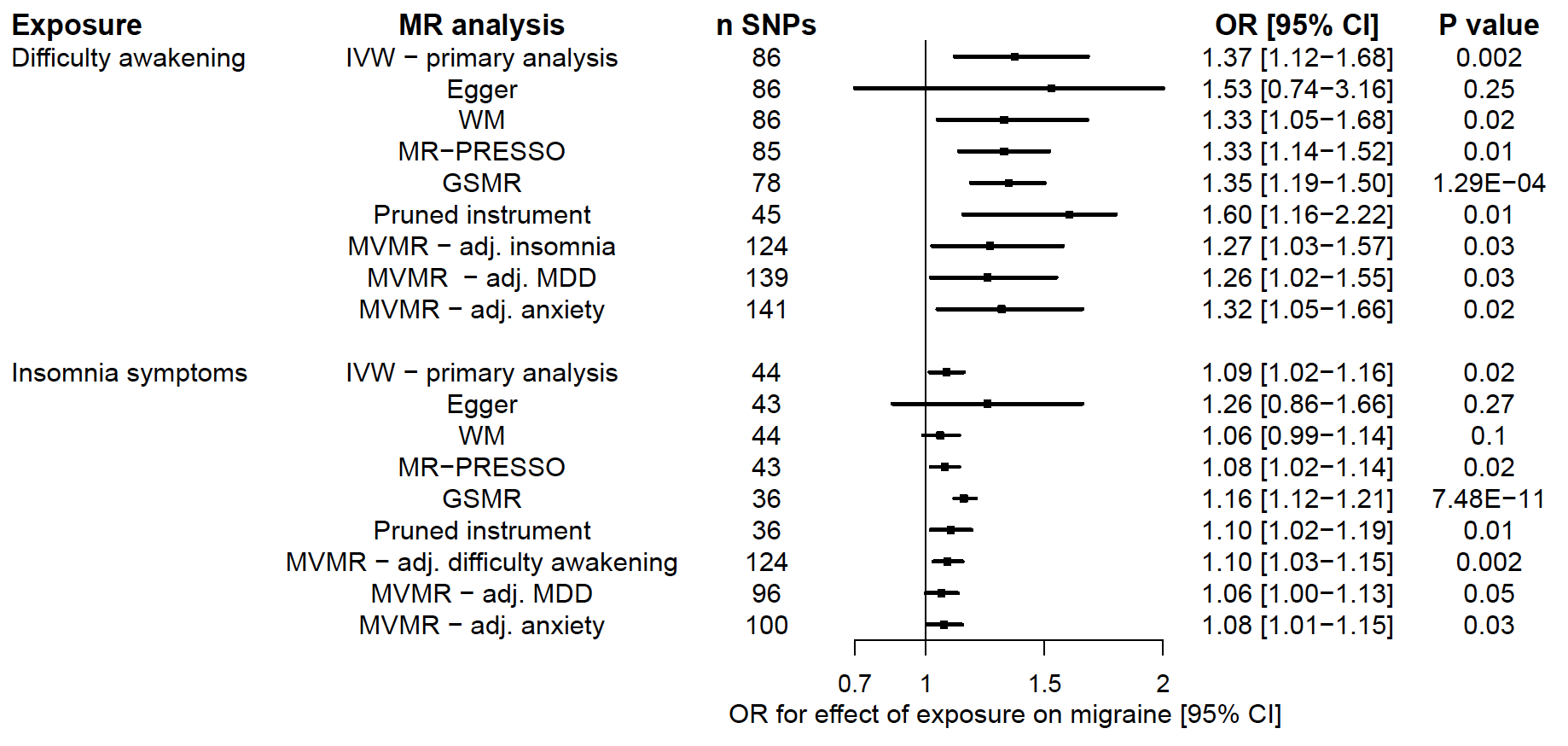
**

**Supplementary Figure 1. Forest plot of two-sample Mendelian randomization sensitivity analyses for the effect of difficulty awakening and liability to insomnia symptoms on risk of migraine (59,674 cases and 316,078 controls).** MR-PRESSO results are shown after removal of the rs324017 outlier variant for insomnia and rs6676685 outlier variant for difficulty awakening. Results displayed from the Egger regression exclude the rs113851554 variant identified in leave-one-out analyses. CI: confidence interval; GSMR: generalized summary data-based MR; IVW: inverse-variance weighted; MDD: major depressive disorder; MVMR: multivariable MR; WM: weighted median.

**
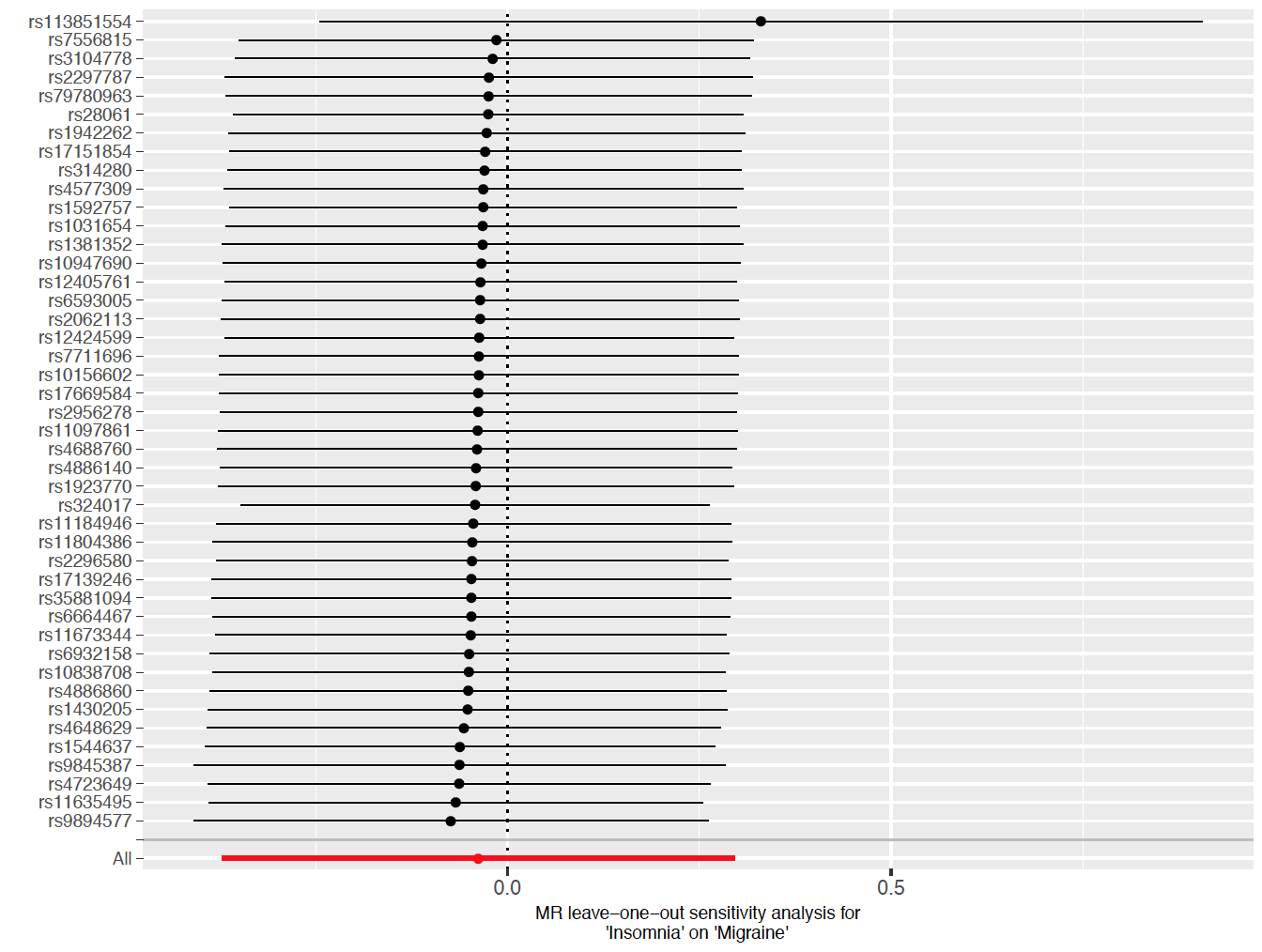
 Supplementary Figure 2. Leave-one-out plot for MR Egger effect of liability to insomnia symptoms on risk of migraine.** The plot demonstrates influence of rs113851554 (in MEIS1) on the MR Egger estimate. Units on the x-axis correspond to the log-odds of migraine per doubling in the odds of insomnia symptoms.


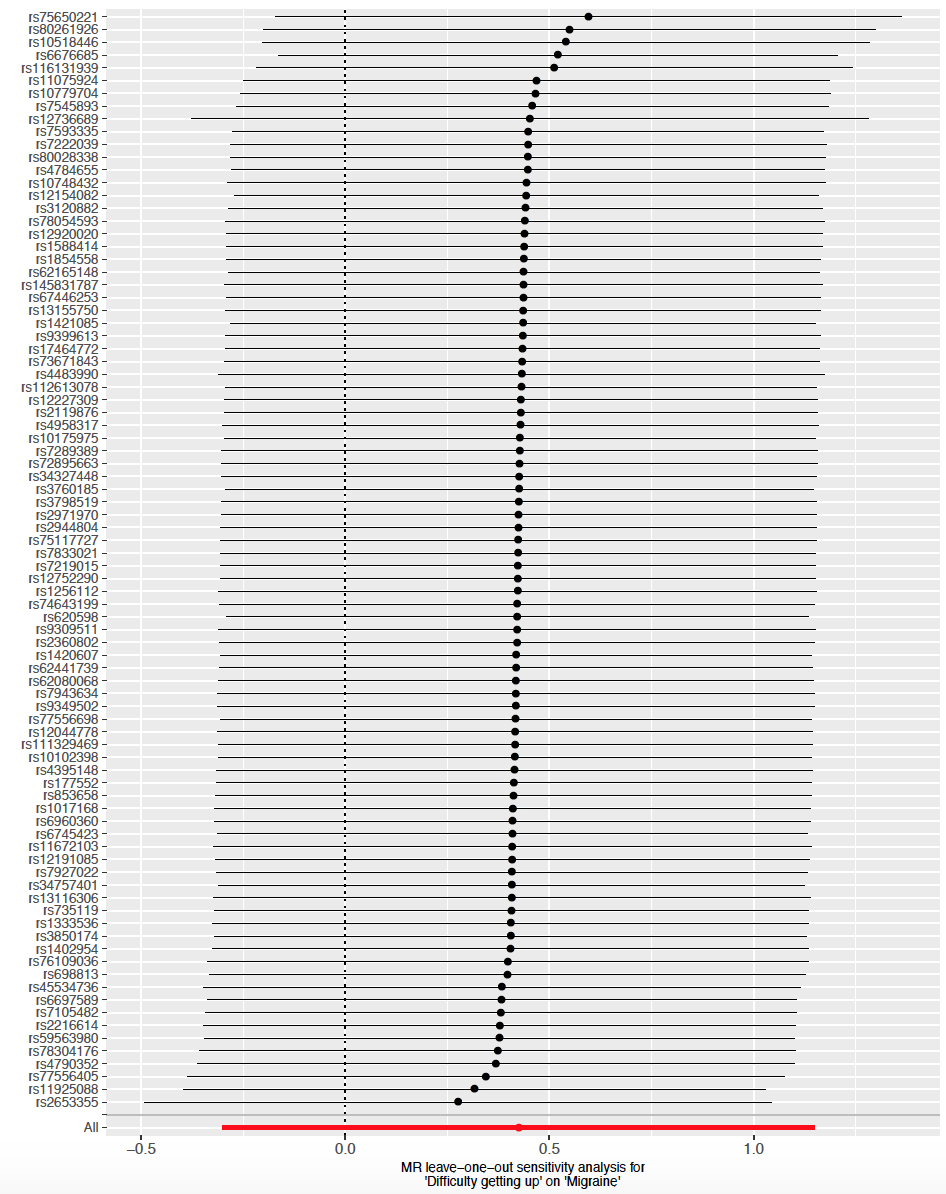


**Supplementary Figure 3. Leave-one-out plot for MR Egger effect of difficulty awakening on risk of migraine.** Units on the x-axis correspond to the log-odds of migraine per unit increase in difficulty awakening.

**
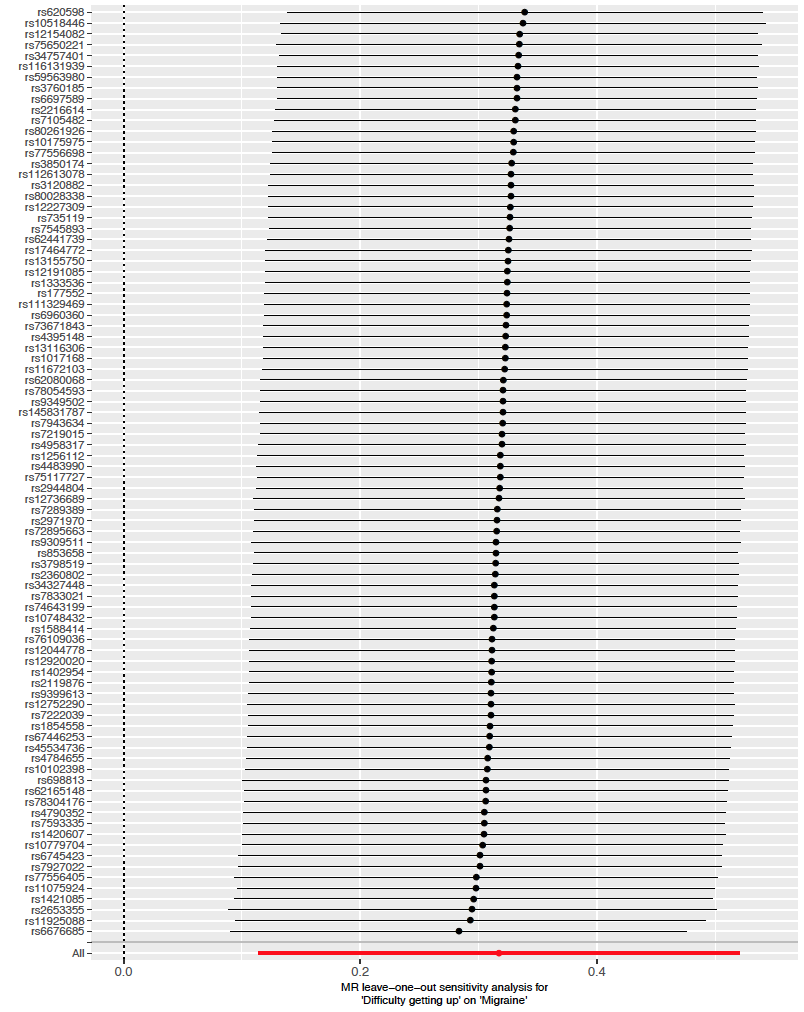
**

**Supplementary Figure 4.** **Leave-one-out MR estimates for the effect of difficulty awakening on risk of migraine.** Units on the x-axis correspond to the log-odds of migraine per unit increase in difficulty awakening on the ordinal scale. The plot reveals no undue influence of any single variant on MR effect estimates. IVW: inverse-variance weighted.

**
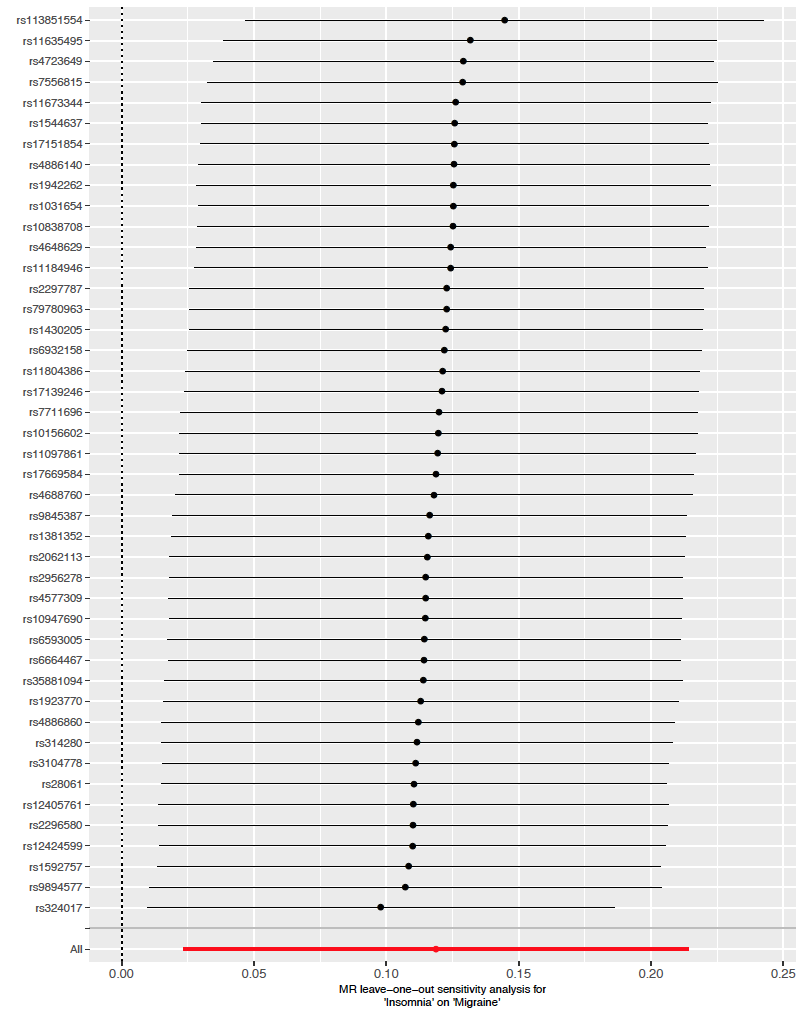
**

**Supplementary Figure 5**. **Leave-one-out MR estimates for the effect of insomnia symptoms on risk of migraine.** Units on the x-axis correspond to the log-odds of migraine per doubling in the odds of insomnia symptoms. The plot reveals no undue influence of any single variant on MR effect estimates.

**Supplementary table 3.** Variants used as genetic instrumental variables for sleep exposures. The raw effects from the ease of awakening GWAS are provided, although these were oriented to difficulty awakening prior to analysis by multiplying effects by -1. DP: diurnal preference; EA: effect allele; OA: other allele; EAF: effect allele frequency; SE: standard error

| **SNP** | **Exposure** | **EA** | **OA** | **EAF** | **Beta** | **SE** |
| --- | --- | --- | --- | --- | --- | --- |
| rs10173260 | Sleep duration | C | T | 0.606235 | 0.0128368 | 0.00231322 |
| rs10483350 | Sleep duration | G | A | 0.195418 | 0.017369 | 0.0028676 |
| rs10761674 | Sleep duration | C | T | 0.477334 | 0.0123329 | 0.00226591 |
| rs1079727 | Sleep duration | C | T | 0.157818 | 0.0182922 | 0.00310347 |
| rs10973207 | Sleep duration | T | G | 0.157677 | 0.0204339 | 0.00312394 |
| rs11039544 | Sleep duration | G | A | 0.837779 | 0.018389 | 0.00307361 |
| rs11067359 | Sleep duration | G | A | 0.748608 | 0.0142791 | 0.0026059 |
| rs11190970 | Sleep duration | G | A | 0.798661 | 0.015379 | 0.00282318 |
| rs112230981 | Sleep duration | A | G | 0.94984 | 0.0315275 | 0.00522787 |
| rs112756201 | Sleep duration | A | G | 0.162484 | 0.0168431 | 0.00306764 |
| rs113113059 | Sleep duration | T | C | 0.78 | 0.0161406 | 0.00273732 |
| rs11567976 | Sleep duration | T | C | 0.570908 | 0.0128042 | 0.00228516 |
| rs11621908 | Sleep duration | C | T | 0.917141 | 0.0240951 | 0.00416298 |
| rs11643715 | Sleep duration | G | C | 0.290942 | 0.013895 | 0.00249658 |
| rs11885663 | Sleep duration | T | C | 0.247809 | 0.0162176 | 0.00261811 |
| rs12215241 | Sleep duration | G | A | 0.772551 | 0.0167219 | 0.00269917 |
| rs12246842 | Sleep duration | A | G | 0.459815 | 0.0133949 | 0.00227425 |
| rs12567114 | Sleep duration | A | G | 0.275802 | 0.0148307 | 0.0025403 |
| rs12607679 | Sleep duration | T | C | 0.737717 | 0.0201387 | 0.00259284 |
| rs12611523 | Sleep duration | A | G | 0.545244 | 0.0126347 | 0.00227627 |
| rs12791153 | Sleep duration | T | A | 0.081089 | 0.0235481 | 0.0042169 |
| rs13088093 | Sleep duration | G | T | 0.336317 | 0.0162722 | 0.00240235 |
| rs13109404 | Sleep duration | T | G | 0.928024 | 0.0312035 | 0.00440829 |
| rs147114641 | Sleep duration | C | A | 0.773722 | 0.0159801 | 0.00270379 |
| rs151014368 | Sleep duration | A | G | 0.206258 | 0.0160924 | 0.00281958 |
| rs1517572 | Sleep duration | C | A | 0.580536 | 0.0146443 | 0.00229467 |
| rs1553132 | Sleep duration | G | A | 0.258433 | 0.0145068 | 0.00258447 |
| rs1724390 | Sleep duration | C | A | 0.773652 | 0.0161086 | 0.0027054 |
| rs17337790 | Sleep duration | T | A | 0.740087 | 0.0146373 | 0.00258376 |
| rs17427571 | Sleep duration | A | G | 0.684313 | 0.0138255 | 0.00243544 |
| rs174560 | Sleep duration | C | T | 0.314215 | 0.0135751 | 0.00243675 |
| rs1776776 | Sleep duration | T | C | 0.873832 | 0.019963 | 0.00341071 |
| rs1939455 | Sleep duration | G | T | 0.879446 | 0.0204253 | 0.00356118 |
| rs205024 | Sleep duration | T | C | 0.383735 | 0.0138261 | 0.00232711 |
| rs2072727 | Sleep duration | T | C | 0.43617 | 0.0132425 | 0.00228506 |
| rs2079070 | Sleep duration | C | G | 0.264613 | 0.0175475 | 0.00256638 |
| rs2192528 | Sleep duration | A | G | 0.480065 | 0.0133687 | 0.0022692 |
| rs2231265 | Sleep duration | G | A | 0.772289 | 0.014955 | 0.00269917 |
| rs2683618 | Sleep duration | G | A | 0.554356 | 0.0177716 | 0.00227689 |
| rs3095508 | Sleep duration | C | A | 0.593529 | 0.0153518 | 0.00230437 |
| rs330088 | Sleep duration | C | T | 0.547012 | 0.0144687 | 0.00227678 |
| rs34354917 | Sleep duration | C | A | 0.710472 | 0.0137464 | 0.00250081 |
| rs34731055 | Sleep duration | T | C | 0.18089 | 0.0194603 | 0.00294778 |
| rs35531607 | Sleep duration | C | T | 0.474083 | 0.0128402 | 0.00227278 |
| rs365663 | Sleep duration | A | G | 0.545963 | 0.0146291 | 0.00227857 |
| rs374153 | Sleep duration | C | T | 0.158085 | 0.0176119 | 0.00310308 |
| rs3751046 | Sleep duration | G | A | 0.146493 | 0.0194129 | 0.00320818 |
| rs4128364 | Sleep duration | C | T | 0.339025 | 0.0145951 | 0.00239086 |
| rs4538155 | Sleep duration | T | C | 0.647426 | 0.0129753 | 0.00237383 |
| rs4592416 | Sleep duration | G | A | 0.464407 | 0.0146828 | 0.0022701 |
| rs460692 | Sleep duration | C | T | 0.137484 | 0.0210557 | 0.00333164 |
| rs4767550 | Sleep duration | G | A | 0.414138 | 0.0143001 | 0.0023096 |
| rs55658675 | Sleep duration | C | T | 0.644938 | 0.0131415 | 0.00236892 |
| rs56372231 | Sleep duration | T | C | 0.334093 | 0.0169439 | 0.00239981 |
| rs61796569 | Sleep duration | T | C | 0.269583 | 0.0154442 | 0.00256443 |
| rs61985058 | Sleep duration | T | C | 0.143176 | 0.0185938 | 0.00322893 |
| rs62120041 | Sleep duration | T | C | 0.933902 | 0.0261113 | 0.00457497 |
| rs6575005 | Sleep duration | T | C | 0.757854 | 0.0155637 | 0.00264172 |
| rs7115462 | Sleep duration | A | G | 0.07353 | 0.0265639 | 0.00435799 |
| rs72817946 | Sleep duration | T | C | 0.9111 | 0.0230753 | 0.00398843 |
| rs73219758 | Sleep duration | G | A | 0.708064 | 0.0164012 | 0.00249526 |
| rs7503199 | Sleep duration | C | T | 0.734267 | 0.0147449 | 0.00256383 |
| rs75539574 | Sleep duration | C | A | 0.085792 | 0.0362482 | 0.00406522 |
| rs7556815 | Sleep duration | A | G | 0.219144 | 0.0407248 | 0.00274023 |
| rs75892241 | Sleep duration | C | A | 0.086977 | 0.0359112 | 0.00404818 |
| rs7644809 | Sleep duration | T | C | 0.421606 | 0.0130621 | 0.00230148 |
| rs7915425 | Sleep duration | T | C | 0.174682 | 0.0190638 | 0.00298959 |
| rs7951019 | Sleep duration | G | T | 0.032227 | 0.0368792 | 0.00652085 |
| rs8038326 | Sleep duration | A | G | 0.72691 | 0.0159204 | 0.0025407 |
| rs915416 | Sleep duration | C | G | 0.289947 | 0.0192587 | 0.00249452 |
| rs9345234 | Sleep duration | C | A | 0.578016 | 0.0130117 | 0.00229893 |
| rs9382445 | Sleep duration | T | C | 0.62305 | 0.014536 | 0.00233387 |
| rs9895274 | Sleep duration | C | T | 0.510408 | 0.0133426 | 0.0022678 |
| rs9903973 | Sleep duration | C | T | 0.46702 | 0.0127747 | 0.00227194 |
| rs9940128 | Sleep duration | G | A | 0.577472 | 0.0169521 | 0.00229097 |
| rs10899257 | Long sleep | A | G | 0.144473 | 0.062094186 | 0.011357834 |
| rs117124984 | Long sleep | C | G | 0.78164 | 0.065567022 | 0.009761319 |
| rs147114641 | Long sleep | C | A | 0.77403 | 0.062655756 | 0.009555039 |
| rs17817288 | Long sleep | A | G | 0.518127 | 0.046126499 | 0.008001421 |
| rs3751046 | Long sleep | G | A | 0.147342 | 0.063601471 | 0.01131312 |
| rs67155855 | Long sleep | G | A | 0.797017 | 0.060936794 | 0.010255388 |
| rs6737318 | Long sleep | G | A | 0.221841 | 0.070265311 | 0.009653256 |
| rs75458655 | Long sleep | T | C | 0.022973 | 0.184261941 | 0.026684765 |
| rs10058356 | Morning DP | C | T | 0.301192 | 0.0169817 | 0.00293612 |
| rs10067113 | Morning DP | T | C | 0.619099 | 0.016251 | 0.00277635 |
| rs10149448 | Morning DP | A | G | 0.603599 | 0.0164465 | 0.00275282 |
| rs10175975 | Morning DP | T | C | 0.180474 | 0.0228441 | 0.00351376 |
| rs10193431 | Morning DP | T | C | 0.525892 | 0.0163321 | 0.00268854 |
| rs1027742 | Morning DP | A | G | 0.737765 | 0.0183119 | 0.00306482 |
| rs10280205 | Morning DP | T | C | 0.691748 | 0.0174497 | 0.00291239 |
| rs10402849 | Morning DP | T | C | 0.201353 | 0.0192609 | 0.00335946 |
| rs10495976 | Morning DP | T | A | 0.390221 | 0.0178512 | 0.00276698 |
| rs10520176 | Morning DP | T | C | 0.50187 | 0.0231498 | 0.00268932 |
| rs1053007 | Morning DP | G | A | 0.632478 | 0.0153999 | 0.00278963 |
| rs1064213 | Morning DP | A | G | 0.477688 | 0.019141 | 0.00268602 |
| rs10742179 | Morning DP | A | G | 0.260177 | 0.0182967 | 0.00306465 |
| rs10747046 | Morning DP | G | A | 0.337292 | 0.0170793 | 0.00285233 |
| rs10758942 | Morning DP | A | G | 0.357303 | 0.0167001 | 0.00280506 |
| rs10772978 | Morning DP | T | A | 0.644659 | 0.0164843 | 0.00280771 |
| rs10818834 | Morning DP | T | C | 0.732704 | 0.0188673 | 0.00304915 |
| rs10823233 | Morning DP | C | T | 0.547887 | 0.0151686 | 0.00270212 |
| rs10976942 | Morning DP | A | C | 0.08298 | 0.0282753 | 0.00487069 |
| rs10988239 | Morning DP | C | T | 0.48728 | 0.0186334 | 0.00273304 |
| rs11032362 | Morning DP | A | G | 0.090763 | 0.0395538 | 0.00467579 |
| rs11083105 | Morning DP | G | A | 0.594686 | 0.0184584 | 0.00275495 |
| rs1113295 | Morning DP | T | G | 0.440869 | 0.0156591 | 0.00270408 |
| rs11152350 | Morning DP | C | A | 0.528998 | 0.0163037 | 0.00269517 |
| rs111626088 | Morning DP | A | C | 0.075622 | 0.0313664 | 0.00509283 |
| rs11165756 | Morning DP | C | A | 0.809522 | 0.0215941 | 0.00341918 |
| rs11183201 | Morning DP | C | T | 0.50779 | 0.0179025 | 0.0026954 |
| rs11227452 | Morning DP | A | G | 0.171642 | 0.0194632 | 0.00358768 |
| rs113387037 | Morning DP | G | A | 0.9386 | 0.0330468 | 0.00569033 |
| rs113389356 | Morning DP | G | A | 0.883946 | 0.0229579 | 0.00421963 |
| rs113441737 | Morning DP | A | G | 0.078412 | 0.0279515 | 0.00510592 |
| rs113851554 | Morning DP | G | T | 0.942753 | 0.0357353 | 0.00598996 |
| rs114848860 | Morning DP | T | A | 0.024463 | 0.0555837 | 0.00872612 |
| rs11587758 | Morning DP | A | G | 0.395515 | 0.0241103 | 0.0027375 |
| rs11588913 | Morning DP | G | A | 0.601681 | 0.0154525 | 0.00273856 |
| rs116298301 | Morning DP | T | C | 0.028034 | 0.0664317 | 0.00832197 |
| rs11641239 | Morning DP | T | C | 0.288572 | 0.0172486 | 0.00296753 |
| rs11670534 | Morning DP | C | T | 0.834382 | 0.0209755 | 0.00362802 |
| rs11677484 | Morning DP | T | G | 0.255695 | 0.0185564 | 0.00309256 |
| rs11712056 | Morning DP | T | C | 0.556296 | 0.0200896 | 0.00270384 |
| rs11786306 | Morning DP | C | G | 0.353978 | 0.0183309 | 0.00283083 |
| rs12055234 | Morning DP | A | G | 0.328358 | 0.0188521 | 0.00286239 |
| rs12140153 | Morning DP | G | T | 0.904737 | 0.0340109 | 0.00468803 |
| rs12432176 | Morning DP | A | C | 0.379295 | 0.0161131 | 0.00278357 |
| rs12462111 | Morning DP | C | T | 0.535169 | 0.0158171 | 0.00273479 |
| rs12541362 | Morning DP | T | A | 0.350242 | 0.0213568 | 0.00282446 |
| rs12713014 | Morning DP | A | G | 0.942045 | 0.0353634 | 0.00579576 |
| rs12798330 | Morning DP | A | G | 0.962009 | 0.0388668 | 0.00702635 |
| rs12927162 | Morning DP | A | G | 0.721593 | 0.0286498 | 0.00300067 |
| rs12969848 | Morning DP | T | C | 0.529859 | 0.0218632 | 0.00270301 |
| rs13011556 | Morning DP | G | C | 0.238596 | 0.0227424 | 0.00316075 |
| rs13059636 | Morning DP | G | A | 0.472049 | 0.0182698 | 0.00270236 |
| rs13065394 | Morning DP | G | T | 0.711537 | 0.017504 | 0.00296404 |
| rs13255030 | Morning DP | A | G | 0.585145 | 0.0162198 | 0.00273214 |
| rs13269289 | Morning DP | A | G | 0.326935 | 0.016463 | 0.00289266 |
| rs139911 | Morning DP | C | T | 0.423865 | 0.0233915 | 0.00273231 |
| rs1401315 | Morning DP | G | A | 0.10495 | 0.0279789 | 0.00442923 |
| rs1421085 | Morning DP | C | T | 0.403118 | 0.0275621 | 0.00274075 |
| rs149611468 | Morning DP | T | C | 0.988036 | 0.0776374 | 0.0125983 |
| rs1555531 | Morning DP | A | G | 0.110785 | 0.0283742 | 0.00426902 |
| rs17161045 | Morning DP | T | C | 0.630545 | 0.0206395 | 0.0028029 |
| rs17267683 | Morning DP | C | G | 0.76125 | 0.0197414 | 0.00316651 |
| rs17374439 | Morning DP | T | C | 0.196665 | 0.0340816 | 0.0033738 |
| rs17399554 | Morning DP | A | G | 0.254397 | 0.017892 | 0.00308801 |
| rs17448682 | Morning DP | T | C | 0.231952 | 0.0221528 | 0.00318247 |
| rs17575798 | Morning DP | G | A | 0.807172 | 0.0225553 | 0.00339377 |
| rs17604349 | Morning DP | G | A | 0.820797 | 0.0257422 | 0.00351443 |
| rs17682747 | Morning DP | A | G | 0.232314 | 0.0184215 | 0.00319016 |
| rs1800828 | Morning DP | C | G | 0.746407 | 0.0176235 | 0.00308462 |
| rs184033703 | Morning DP | A | G | 0.057979 | 0.0377937 | 0.00576165 |
| rs1871516 | Morning DP | T | C | 0.669022 | 0.0158288 | 0.00285183 |
| rs1931814 | Morning DP | A | G | 0.478075 | 0.0153877 | 0.00268976 |
| rs1947198 | Morning DP | T | C | 0.122155 | 0.0241964 | 0.00409707 |
| rs1952923 | Morning DP | G | A | 0.260667 | 0.016877 | 0.00305963 |
| rs197273 | Morning DP | A | G | 0.470208 | 0.0158713 | 0.00269376 |
| rs202157 | Morning DP | C | T | 0.298716 | 0.0255486 | 0.0029433 |
| rs2072727 | Morning DP | T | C | 0.435988 | 0.016352 | 0.00271514 |
| rs2102506 | Morning DP | G | A | 0.360336 | 0.0174762 | 0.00281907 |
| rs2189008 | Morning DP | T | C | 0.492589 | 0.0155929 | 0.00269632 |
| rs2239626 | Morning DP | C | T | 0.304589 | 0.0202758 | 0.00292696 |
| rs226090 | Morning DP | C | T | 0.168356 | 0.0208783 | 0.00359523 |
| rs231398 | Morning DP | G | A | 0.837853 | 0.0207867 | 0.00367183 |
| rs2364972 | Morning DP | G | A | 0.462754 | 0.0162127 | 0.00270183 |
| rs2518022 | Morning DP | T | C | 0.0836026 | 0.0403075 | 0.00485089 |
| rs2545799 | Morning DP | G | T | 0.546139 | 0.0148163 | 0.00271089 |
| rs2550298 | Morning DP | C | T | 0.622827 | 0.0239989 | 0.0027767 |
| rs2580160 | Morning DP | A | G | 0.552952 | 0.0153499 | 0.00273342 |
| rs2653349 | Morning DP | A | G | 0.214552 | 0.0387249 | 0.00327448 |
| rs2706762 | Morning DP | C | T | 0.850258 | 0.0219218 | 0.00375432 |
| rs2712056 | Morning DP | T | C | 0.184664 | 0.022175 | 0.00345992 |
| rs2744915 | Morning DP | G | A | 0.61016 | 0.014967 | 0.00276129 |
| rs28380327 | Morning DP | A | T | 0.629124 | 0.0205097 | 0.00277698 |
| rs28634184 | Morning DP | C | T | 0.749137 | 0.0176363 | 0.00310689 |
| rs2881955 | Morning DP | T | C | 0.27878 | 0.0161216 | 0.00300229 |
| rs295268 | Morning DP | C | T | 0.255675 | 0.0165883 | 0.00307939 |
| rs2968511 | Morning DP | G | C | 0.304708 | 0.0174599 | 0.00291928 |
| rs2971970 | Morning DP | T | G | 0.218075 | 0.0188272 | 0.00325601 |
| rs308521 | Morning DP | T | C | 0.602627 | 0.0199718 | 0.00275427 |
| rs3100052 | Morning DP | A | G | 0.387351 | 0.0170795 | 0.00276058 |
| rs3168135 | Morning DP | G | A | 0.760045 | 0.0207725 | 0.0031468 |
| rs35653190 | Morning DP | C | T | 0.771675 | 0.0182444 | 0.00322514 |
| rs35687504 | Morning DP | C | A | 0.809996 | 0.0201251 | 0.00346773 |
| rs359250 | Morning DP | T | G | 0.636336 | 0.0168642 | 0.0027885 |
| rs35935113 | Morning DP | T | C | 0.552691 | 0.0169852 | 0.00270472 |
| rs36055559 | Morning DP | G | A | 0.827204 | 0.0210912 | 0.00372199 |
| rs3760185 | Morning DP | C | T | 0.751745 | 0.0241586 | 0.0031496 |
| rs3808477 | Morning DP | T | C | 0.281399 | 0.0215613 | 0.00299116 |
| rs3808964 | Morning DP | T | G | 0.634729 | 0.0157096 | 0.00279601 |
| rs3850174 | Morning DP | T | A | 0.742891 | 0.0194471 | 0.00309938 |
| rs3857599 | Morning DP | A | C | 0.163038 | 0.0218522 | 0.00363894 |
| rs4237555 | Morning DP | T | C | 0.526954 | 0.0164771 | 0.00269332 |
| rs4239386 | Morning DP | T | A | 0.665104 | 0.0218962 | 0.00285562 |
| rs4241964 | Morning DP | G | T | 0.475089 | 0.020735 | 0.00270449 |
| rs4316965 | Morning DP | C | T | 0.626252 | 0.0159238 | 0.00278976 |
| rs4321976 | Morning DP | T | C | 0.77856 | 0.0232959 | 0.00324034 |
| rs4339281 | Morning DP | A | G | 0.873018 | 0.0223188 | 0.00404047 |
| rs4350910 | Morning DP | G | A | 0.117523 | 0.0257358 | 0.00419108 |
| rs4490386 | Morning DP | G | A | 0.692499 | 0.0180125 | 0.00291001 |
| rs4654880 | Morning DP | C | A | 0.395694 | 0.0198985 | 0.00275349 |
| rs4714475 | Morning DP | G | T | 0.754058 | 0.0190271 | 0.00317856 |
| rs4729303 | Morning DP | T | C | 0.813151 | 0.0246891 | 0.00344383 |
| rs4838161 | Morning DP | T | G | 0.560809 | 0.0161104 | 0.00270516 |
| rs4852864 | Morning DP | C | T | 0.407109 | 0.0149792 | 0.00272573 |
| rs4912145 | Morning DP | C | T | 0.34083 | 0.0185204 | 0.00285587 |
| rs4936291 | Morning DP | G | A | 0.389252 | 0.0179295 | 0.00283312 |
| rs4948547 | Morning DP | C | A | 0.255795 | 0.0190042 | 0.00307909 |
| rs4949980 | Morning DP | G | A | 0.067607 | 0.0321893 | 0.00539402 |
| rs509476 | Morning DP | T | C | 0.0301569 | 0.1137 | 0.0078575 |
| rs520954 | Morning DP | G | A | 0.327282 | 0.0235996 | 0.00285975 |
| rs521977 | Morning DP | T | G | 0.322419 | 0.0159787 | 0.00286438 |
| rs55947153 | Morning DP | T | A | 0.588479 | 0.0161167 | 0.00273752 |
| rs56049037 | Morning DP | G | A | 0.712303 | 0.0207567 | 0.00297982 |
| rs56947091 | Morning DP | T | C | 0.511326 | 0.0191881 | 0.00268737 |
| rs57435966 | Morning DP | C | T | 0.912868 | 0.0549152 | 0.0047647 |
| rs577924 | Morning DP | T | C | 0.535103 | 0.0155309 | 0.00270069 |
| rs59986227 | Morning DP | G | C | 0.25779 | 0.0178401 | 0.00310217 |
| rs60616179 | Morning DP | A | G | 0.945102 | 0.0375809 | 0.00594669 |
| rs6131942 | Morning DP | G | A | 0.580035 | 0.0175437 | 0.00273235 |
| rs62082401 | Morning DP | G | C | 0.19099 | 0.0256287 | 0.00342253 |
| rs62182135 | Morning DP | C | A | 0.66833 | 0.0179439 | 0.00284795 |
| rs62553781 | Morning DP | C | T | 0.965171 | 0.0504004 | 0.00734656 |
| rs640245 | Morning DP | T | C | 0.483356 | 0.0182658 | 0.00269872 |
| rs6442446 | Morning DP | A | G | 0.290534 | 0.0174738 | 0.00297308 |
| rs6452786 | Morning DP | G | T | 0.74731 | 0.0212678 | 0.00308986 |
| rs6477309 | Morning DP | T | C | 0.665392 | 0.0174399 | 0.00286276 |
| rs651001 | Morning DP | G | A | 0.569703 | 0.0155881 | 0.0027154 |
| rs6577620 | Morning DP | C | T | 0.547454 | 0.0170777 | 0.00271211 |
| rs6658041 | Morning DP | A | G | 0.59992 | 0.0152482 | 0.00274124 |
| rs66710942 | Morning DP | T | C | 0.593057 | 0.0174576 | 0.0027476 |
| rs6744983 | Morning DP | T | G | 0.377534 | 0.0162918 | 0.00276872 |
| rs6773311 | Morning DP | T | G | 0.674316 | 0.0166158 | 0.00286155 |
| rs67988891 | Morning DP | G | C | 0.318795 | 0.0232401 | 0.00288939 |
| rs6967481 | Morning DP | T | C | 0.496563 | 0.0206433 | 0.00269925 |
| rs698015 | Morning DP | T | C | 0.647493 | 0.0161876 | 0.0028566 |
| rs7144406 | Morning DP | A | G | 0.790526 | 0.0184778 | 0.00331646 |
| rs7148842 | Morning DP | C | T | 0.611932 | 0.0153777 | 0.00278312 |
| rs72632979 | Morning DP | A | G | 0.82849 | 0.021581 | 0.00357606 |
| rs72720396 | Morning DP | G | A | 0.230344 | 0.0271261 | 0.00318521 |
| rs72829706 | Morning DP | A | G | 0.960574 | 0.0407112 | 0.00693598 |
| rs72966564 | Morning DP | C | T | 0.750914 | 0.0174371 | 0.00312692 |
| rs72992015 | Morning DP | T | C | 0.209605 | 0.0187449 | 0.00330925 |
| rs7304278 | Morning DP | G | A | 0.725152 | 0.020255 | 0.00302264 |
| rs7313852 | Morning DP | G | A | 0.564964 | 0.029649 | 0.00271587 |
| rs741334 | Morning DP | A | G | 0.711693 | 0.0164509 | 0.0029772 |
| rs74357745 | Morning DP | A | G | 0.878807 | 0.026546 | 0.00412156 |
| rs75120545 | Morning DP | T | C | 0.030179 | 0.0613917 | 0.00828587 |
| rs7547493 | Morning DP | G | A | 0.178016 | 0.0376299 | 0.00350319 |
| rs75650221 | Morning DP | T | C | 0.038378 | 0.0421207 | 0.00699783 |
| rs762995 | Morning DP | G | A | 0.534757 | 0.0155872 | 0.00269633 |
| rs769066 | Morning DP | C | T | 0.183481 | 0.0219436 | 0.00348289 |
| rs7691121 | Morning DP | C | G | 0.767817 | 0.0220069 | 0.00317772 |
| rs7701529 | Morning DP | T | A | 0.762136 | 0.0188448 | 0.00317247 |
| rs77384811 | Morning DP | G | C | 0.843254 | 0.0247483 | 0.00372778 |
| rs7743404 | Morning DP | C | A | 0.541815 | 0.016019 | 0.002696 |
| rs77655131 | Morning DP | C | T | 0.874196 | 0.0273926 | 0.00408599 |
| rs778138 | Morning DP | C | A | 0.351139 | 0.0205691 | 0.00280797 |
| rs78095690 | Morning DP | C | T | 0.435624 | 0.0163477 | 0.00272137 |
| rs78118301 | Morning DP | T | A | 0.010468 | 0.107046 | 0.0132716 |
| rs7827072 | Morning DP | G | A | 0.523042 | 0.015179 | 0.00269079 |
| rs786406 | Morning DP | G | A | 0.702127 | 0.0235784 | 0.00293536 |
| rs7943634 | Morning DP | C | T | 0.690715 | 0.016455 | 0.00291003 |
| rs7959983 | Morning DP | C | T | 0.404082 | 0.0195258 | 0.00272849 |
| rs80097534 | Morning DP | G | T | 0.901458 | 0.0281025 | 0.00455453 |
| rs8063159 | Morning DP | A | C | 0.389082 | 0.0192263 | 0.00275742 |
| rs9348050 | Morning DP | T | C | 0.48949 | 0.016638 | 0.00269048 |
| rs9364767 | Morning DP | T | G | 0.446852 | 0.0158538 | 0.00269933 |
| rs9395520 | Morning DP | T | C | 0.305433 | 0.0231272 | 0.00291912 |
| rs9521184 | Morning DP | T | C | 0.48682 | 0.0148855 | 0.00270389 |
| rs9573971 | Morning DP | A | G | 0.9661 | 0.0727759 | 0.00743443 |
| rs957501 | Morning DP | T | A | 0.336064 | 0.0154211 | 0.00284661 |
| rs9597250 | Morning DP | C | A | 0.810212 | 0.0213068 | 0.00343801 |
| rs960783 | Morning DP | C | T | 0.955376 | 0.060508 | 0.00654133 |
| rs9636202 | Morning DP | G | A | 0.733565 | 0.0183734 | 0.00305534 |
| rs975025 | Morning DP | C | T | 0.922916 | 0.0321062 | 0.00502525 |
| rs9831488 | Morning DP | G | A | 0.350467 | 0.0183592 | 0.00284523 |
| rs9932577 | Morning DP | C | A | 0.495067 | 0.0166195 | 0.00271482 |
| rs9964420 | Morning DP | C | A | 0.695492 | 0.0284801 | 0.00293643 |
| rs1001817 | Napping | C | T | 0.507212 | 0.00775426 | 0.00121418 |
| rs10149986 | Napping | G | T | 0.185198 | 0.0109384 | 0.00156608 |
| rs10152428 | Napping | C | G | 0.729765 | 0.00780688 | 0.00136996 |
| rs10257273 | Napping | A | T | 0.85069 | 0.0107366 | 0.00170413 |
| rs10737380 | Napping | C | A | 0.676036 | 0.00711351 | 0.00129196 |
| rs10811438 | Napping | G | C | 0.60658 | 0.00721671 | 0.0012428 |
| rs10835420 | Napping | T | A | 0.750826 | 0.00889802 | 0.00140206 |
| rs10840017 | Napping | A | G | 0.767369 | 0.00894142 | 0.00148101 |
| rs10852455 | Napping | G | A | 0.286798 | 0.0079181 | 0.00134214 |
| rs10875606 | Napping | C | A | 0.315767 | 0.00737707 | 0.0013077 |
| rs10875622 | Napping | A | G | 0.575365 | 0.0104621 | 0.0012291 |
| rs11071755 | Napping | G | A | 0.574922 | 0.00711401 | 0.00122819 |
| rs11125776 | Napping | T | G | 0.854478 | 0.0116502 | 0.00172495 |
| rs11224896 | Napping | T | C | 0.889747 | 0.0111605 | 0.00193872 |
| rs112520848 | Napping | C | G | 0.385884 | 0.00702449 | 0.00125191 |
| rs11258652 | Napping | C | A | 0.763185 | 0.0104004 | 0.00142918 |
| rs112997627 | Napping | T | G | 0.77804 | 0.0210273 | 0.00146713 |
| rs115374417 | Napping | G | A | 0.106012 | 0.0137075 | 0.00237326 |
| rs11615756 | Napping | T | C | 0.404611 | 0.0182673 | 0.00123883 |
| rs11860072 | Napping | C | T | 0.552791 | 0.00897773 | 0.00123952 |
| rs11967137 | Napping | A | G | 0.805519 | 0.00927928 | 0.00153285 |
| rs12031519 | Napping | A | G | 0.888258 | 0.0112838 | 0.00194275 |
| rs12140153 | Napping | G | T | 0.904603 | 0.0247147 | 0.00211564 |
| rs12193281 | Napping | T | C | 0.66958 | 0.00875849 | 0.00129144 |
| rs12346996 | Napping | T | C | 0.271929 | 0.00796106 | 0.00136459 |
| rs12451365 | Napping | C | T | 0.204638 | 0.010628 | 0.00150388 |
| rs12497609 | Napping | T | G | 0.162789 | 0.00913115 | 0.00164197 |
| rs12657723 | Napping | T | C | 0.321683 | 0.00818319 | 0.00129849 |
| rs13033444 | Napping | G | A | 0.282805 | 0.00974179 | 0.00135018 |
| rs13150944 | Napping | G | A | 0.689386 | 0.00860076 | 0.0013218 |
| rs13263535 | Napping | G | T | 0.531464 | 0.00687135 | 0.00121641 |
| rs13284688 | Napping | C | T | 0.206572 | 0.0150717 | 0.00149894 |
| rs140506252 | Napping | A | T | 0.977522 | 0.0227261 | 0.0040886 |
| rs1415218 | Napping | T | C | 0.281328 | 0.00820697 | 0.00135243 |
| rs1426907 | Napping | A | G | 0.478135 | 0.00665598 | 0.00121814 |
| rs1477151 | Napping | G | A | 0.484736 | 0.00691772 | 0.00121492 |
| rs1601440 | Napping | C | T | 0.278622 | 0.00912653 | 0.00135575 |
| rs17049683 | Napping | G | A | 0.333319 | 0.00795171 | 0.00129066 |
| rs17158413 | Napping | A | G | 0.237279 | 0.00925014 | 0.00142955 |
| rs17265513 | Napping | C | T | 0.199401 | 0.00913188 | 0.00151965 |
| rs174541 | Napping | C | T | 0.360451 | 0.00988214 | 0.00126293 |
| rs17502738 | Napping | T | C | 0.804111 | 0.00870459 | 0.00153004 |
| rs186099 | Napping | T | A | 0.605783 | 0.00786638 | 0.00124267 |
| rs1883048 | Napping | C | T | 0.523561 | 0.00787143 | 0.00122313 |
| rs1931175 | Napping | G | C | 0.383828 | 0.00776325 | 0.00124756 |
| rs1968557 | Napping | C | T | 0.505765 | 0.00769572 | 0.0012147 |
| rs2195272 | Napping | T | G | 0.329132 | 0.0101237 | 0.00129125 |
| rs224111 | Napping | G | A | 0.612306 | 0.00798411 | 0.00124912 |
| rs2250377 | Napping | A | G | 0.338883 | 0.0131561 | 0.00127975 |
| rs2284015 | Napping | G | C | 0.259172 | 0.00771167 | 0.00138662 |
| rs2370926 | Napping | T | C | 0.633526 | 0.00813213 | 0.00126359 |
| rs2431108 | Napping | C | T | 0.328723 | 0.0129827 | 0.00129028 |
| rs253666 | Napping | A | G | 0.764938 | 0.0081271 | 0.0014309 |
| rs2653349 | Napping | A | G | 0.214777 | 0.0165942 | 0.00147827 |
| rs2699869 | Napping | A | C | 0.454128 | 0.00678084 | 0.00121774 |
| rs271057 | Napping | C | T | 0.248427 | 0.00866783 | 0.00140414 |
| rs2769916 | Napping | A | G | 0.688617 | 0.00875328 | 0.00131344 |
| rs2786547 | Napping | C | T | 0.823104 | 0.0107673 | 0.00158775 |
| rs2836912 | Napping | A | G | 0.311695 | 0.00729991 | 0.00131115 |
| rs2861805 | Napping | A | G | 0.540036 | 0.0093112 | 0.00122068 |
| rs2893323 | Napping | A | G | 0.354058 | 0.0069416 | 0.00126675 |
| rs2943023 | Napping | C | T | 0.580197 | 0.00710422 | 0.00123055 |
| rs301817 | Napping | A | C | 0.5894 | 0.00734402 | 0.00123666 |
| rs34262487 | Napping | C | A | 0.927997 | 0.0145107 | 0.00235463 |
| rs34728579 | Napping | C | T | 0.200202 | 0.00839996 | 0.00152087 |
| rs35011311 | Napping | G | T | 0.734419 | 0.009082 | 0.00138249 |
| rs35039375 | Napping | G | A | 0.091832 | 0.0136431 | 0.00210973 |
| rs350785 | Napping | T | C | 0.115476 | 0.0126479 | 0.00190975 |
| rs35144585 | Napping | T | A | 0.870708 | 0.0103105 | 0.00182249 |
| rs351776 | Napping | C | A | 0.549518 | 0.00755367 | 0.00122072 |
| rs35851551 | Napping | A | G | 0.898496 | 0.011194 | 0.00202654 |
| rs3810484 | Napping | A | G | 0.556304 | 0.00684147 | 0.001222 |
| rs388016 | Napping | A | G | 0.666763 | 0.00738134 | 0.00128736 |
| rs3935190 | Napping | A | G | 0.536252 | 0.00801432 | 0.00122354 |
| rs3986805 | Napping | G | A | 0.414592 | 0.00719435 | 0.00123765 |
| rs41313250 | Napping | A | G | 0.879238 | 0.013072 | 0.00191501 |
| rs4236060 | Napping | T | C | 0.272281 | 0.00882528 | 0.00137545 |
| rs4356873 | Napping | C | T | 0.239996 | 0.00775689 | 0.00142205 |
| rs4357022 | Napping | G | T | 0.464156 | 0.00682396 | 0.00121553 |
| rs4604518 | Napping | G | A | 0.546474 | 0.00691205 | 0.00122091 |
| rs4692709 | Napping | C | T | 0.454598 | 0.00723975 | 0.00122517 |
| rs4793173 | Napping | A | C | 0.37067 | 0.00803948 | 0.00125702 |
| rs4983329 | Napping | A | C | 0.555832 | 0.00767251 | 0.0012207 |
| rs56180058 | Napping | C | T | 0.839025 | 0.00942616 | 0.00165866 |
| rs60222088 | Napping | C | A | 0.852733 | 0.0112358 | 0.00172293 |
| rs60920123 | Napping | G | A | 0.566808 | 0.00763778 | 0.00122697 |
| rs614987 | Napping | C | A | 0.614205 | 0.0110217 | 0.00124925 |
| rs62189006 | Napping | A | G | 0.907199 | 0.0120943 | 0.0020982 |
| rs62560863 | Napping | T | C | 0.100316 | 0.0113172 | 0.0020241 |
| rs6919087 | Napping | T | G | 0.6876 | 0.0107806 | 0.0013114 |
| rs72715294 | Napping | A | T | 0.248388 | 0.00958899 | 0.00140295 |
| rs73817091 | Napping | T | C | 0.042467 | 0.0165127 | 0.00302224 |
| rs7422655 | Napping | C | T | 0.263115 | 0.00783348 | 0.00137827 |
| rs7524405 | Napping | C | A | 0.374865 | 0.00715209 | 0.00125277 |
| rs75932578 | Napping | T | C | 0.215994 | 0.00904281 | 0.0014763 |
| rs76824303 | Napping | A | C | 0.900584 | 0.0117977 | 0.00207749 |
| rs7708715 | Napping | C | A | 0.535451 | 0.00764452 | 0.00121661 |
| rs77154532 | Napping | A | G | 0.641674 | 0.00769324 | 0.0012705 |
| rs7743188 | Napping | C | T | 0.903761 | 0.0128648 | 0.00206234 |
| rs7814873 | Napping | C | T | 0.382994 | 0.00720972 | 0.00126385 |
| rs785145 | Napping | G | T | 0.431455 | 0.0069889 | 0.00122527 |
| rs80163246 | Napping | C | T | 0.117455 | 0.0119129 | 0.00188438 |
| rs8092870 | Napping | T | C | 0.409825 | 0.00699531 | 0.00125126 |
| rs908442 | Napping | A | T | 0.591346 | 0.0102781 | 0.00123558 |
| rs910187 | Napping | G | A | 0.626033 | 0.00731754 | 0.00125594 |
| rs9287862 | Napping | C | T | 0.0867737 | 0.0122526 | 0.00215162 |
| rs9309116 | Napping | T | C | 0.652831 | 0.00737914 | 0.00127277 |
| rs9389556 | Napping | G | C | 0.260947 | 0.00841517 | 0.00138285 |
| rs9460110 | Napping | C | T | 0.369782 | 0.00735568 | 0.00125751 |
| rs962247 | Napping | G | A | 0.523199 | 0.00796355 | 0.00122493 |
| rs971415 | Napping | A | G | 0.877622 | 0.0110652 | 0.00185108 |
| rs9883093 | Napping | G | T | 0.633225 | 0.007341 | 0.00125779 |
| rs9939355 | Napping | C | T | 0.442329 | 0.00726404 | 0.00122438 |
| rs9965170 | Napping | G | A | 0.576607 | 0.0136374 | 0.00122874 |
| rs9998136 | Napping | G | C | 0.74725 | 0.00881596 | 0.00140269 |
| rs11763750 | Short sleep | G | A | 0.814346 | 0.037674419 | 0.006444242 |
| rs1229762 | Short sleep | T | C | 0.664501 | 0.037815928 | 0.005310182 |
| rs12518468 | Short sleep | C | T | 0.328456 | 0.030741553 | 0.005333271 |
| rs12567114 | Short sleep | G | A | 0.7246 | 0.033037934 | 0.005624961 |
| rs12661667 | Short sleep | T | C | 0.263495 | 0.031456623 | 0.005679026 |
| rs12963463 | Short sleep | C | T | 0.299425 | 0.037163177 | 0.005537621 |
| rs13107325 | Short sleep | T | C | 0.074528 | 0.069308803 | 0.009549928 |
| rs1380703 | Short sleep | G | A | 0.383531 | 0.035334368 | 0.005247707 |
| rs143525179 | Short sleep | T | C | 0.258014 | 0.031565797 | 0.00577441 |
| rs1607227 | Short sleep | G | T | 0.704938 | 0.033271172 | 0.005509518 |
| rs17005118 | Short sleep | A | G | 0.264936 | 0.03386244 | 0.00567819 |
| rs17388803 | Short sleep | C | A | 0.105648 | 0.051328956 | 0.00829029 |
| rs2014830 | Short sleep | C | T | 0.698128 | 0.030226603 | 0.005486847 |
| rs205024 | Short sleep | C | T | 0.616724 | 0.028784607 | 0.005150682 |
| rs2820313 | Short sleep | G | A | 0.341112 | 0.031375551 | 0.005277221 |
| rs2863957 | Short sleep | C | A | 0.781508 | 0.053227188 | 0.006064168 |
| rs3776864 | Short sleep | A | C | 0.66721 | 0.029900489 | 0.005324338 |
| rs4585442 | Short sleep | G | A | 0.311023 | 0.0331539 | 0.005413036 |
| rs5757675 | Short sleep | G | T | 0.259528 | 0.033719624 | 0.005740926 |
| rs59779556 | Short sleep | T | G | 0.553827 | 0.028684939 | 0.005045869 |
| rs60882754 | Short sleep | A | T | 0.938985 | 0.059048455 | 0.01045493 |
| rs75539574 | Short sleep | A | C | 0.914664 | 0.058282141 | 0.009021919 |
| rs7939345 | Short sleep | T | G | 0.207569 | 0.033945339 | 0.006173343 |
| rs9321171 | Short sleep | C | T | 0.540122 | 0.027968877 | 0.005046397 |
| rs10156602 | Insomnia symptoms | A | G | 0.638126 | 0.04070934 | 0.005830072 |
| rs1031654 | Insomnia symptoms | C | A | 0.201527 | 0.041313205 | 0.00694533 |
| rs10838708 | Insomnia symptoms | G | A | 0.541245 | 0.033826127 | 0.005637037 |
| rs10947690 | Insomnia symptoms | G | A | 0.261294 | 0.034967593 | 0.006335346 |
| rs11097861 | Insomnia symptoms | G | A | 0.715424 | 0.036880314 | 0.006177617 |
| rs11184946 | Insomnia symptoms | T | C | 0.417223 | 0.035372504 | 0.005634783 |
| rs113851554 | Insomnia symptoms | T | G | 0.057694 | 0.166563607 | 0.012375161 |
| rs11635495 | Insomnia symptoms | C | T | 0.514746 | 0.032743881 | 0.005580757 |
| rs11673344 | Insomnia symptoms | G | A | 0.380184 | 0.03496401 | 0.005752335 |
| rs11804386 | Insomnia symptoms | A | G | 0.332415 | 0.032438164 | 0.005894646 |
| rs12405761 | Insomnia symptoms | A | C | 0.570608 | 0.037195089 | 0.005630153 |
| rs12424599 | Insomnia symptoms | G | A | 0.250221 | 0.03789694 | 0.006429388 |
| rs1381352 | Insomnia symptoms | T | G | 0.431901 | 0.031052174 | 0.005630314 |
| rs1430205 | Insomnia symptoms | T | C | 0.458038 | 0.030968277 | 0.005598631 |
| rs1544637 | Insomnia symptoms | T | C | 0.487502 | 0.030896377 | 0.005583897 |
| rs1592757 | Insomnia symptoms | C | G | 0.356945 | 0.036382931 | 0.005811514 |
| rs17139246 | Insomnia symptoms | C | T | 0.389356 | 0.03130161 | 0.005753905 |
| rs17151854 | Insomnia symptoms | T | G | 0.153806 | 0.043095813 | 0.007740982 |
| rs17669584 | Insomnia symptoms | G | A | 0.19491 | 0.03883442 | 0.007181602 |
| rs1923770 | Insomnia symptoms | T | A | 0.383028 | 0.040107327 | 0.005731683 |
| rs1942262 | Insomnia symptoms | A | G | 0.292258 | 0.044890499 | 0.006130757 |
| rs2062113 | Insomnia symptoms | T | C | 0.429502 | 0.035828462 | 0.005649436 |
| rs2296580 | Insomnia symptoms | G | T | 0.702115 | 0.0415157 | 0.006080032 |
| rs2297787 | Insomnia symptoms | T | A | 0.920084 | 0.061538245 | 0.010311312 |
| rs28061 | Insomnia symptoms | A | G | 0.692042 | 0.033665781 | 0.006060709 |
| rs2956278 | Insomnia symptoms | G | A | 0.214809 | 0.038339251 | 0.006782448 |
| rs3104778 | Insomnia symptoms | A | G | 0.589387 | 0.031419485 | 0.005680314 |
| rs314280 | Insomnia symptoms | G | A | 0.54824 | 0.034777174 | 0.005592834 |
| rs324017 | Insomnia symptoms | A | C | 0.294759 | 0.038954267 | 0.006115258 |
| rs35881094 | Insomnia symptoms | G | T | 0.426213 | 0.044495974 | 0.005642834 |
| rs4577309 | Insomnia symptoms | A | G | 0.468107 | 0.032681804 | 0.005589211 |
| rs4648629 | Insomnia symptoms | A | C | 0.433472 | 0.031013688 | 0.005624436 |
| rs4688760 | Insomnia symptoms | T | C | 0.690062 | 0.043347021 | 0.006036473 |
| rs4723649 | Insomnia symptoms | T | C | 0.419212 | 0.032591023 | 0.005645692 |
| rs4886140 | Insomnia symptoms | G | A | 0.668277 | 0.037207971 | 0.005961312 |
| rs4886860 | Insomnia symptoms | G | C | 0.23362 | 0.045238325 | 0.006573309 |
| rs6593005 | Insomnia symptoms | G | A | 0.740998 | 0.036356763 | 0.006359823 |
| rs6664467 | Insomnia symptoms | G | A | 0.863468 | 0.045351852 | 0.00811413 |
| rs6932158 | Insomnia symptoms | C | T | 0.490999 | 0.0312407 | 0.005572947 |
| rs7556815 | Insomnia symptoms | G | A | 0.779334 | 0.048152174 | 0.006722585 |
| rs7711696 | Insomnia symptoms | T | G | 0.304975 | 0.040971417 | 0.006042512 |
| rs79780963 | Insomnia symptoms | C | T | 0.922415 | 0.059983092 | 0.010382931 |
| rs9845387 | Insomnia symptoms | C | A | 0.959308 | 0.079117955 | 0.01409537 |
| rs9894577 | Insomnia symptoms | A | G | 0.318312 | 0.049773752 | 0.005988124 |
| rs10505911 | Snoring | A | C | 0.219715 | 0.02914398 | 0.005299089 |
| rs10765567 | Snoring | T | A | 0.37293 | 0.025248908 | 0.004545529 |
| rs10799070 | Snoring | C | T | 0.36199 | 0.025183944 | 0.00460068 |
| rs10844665 | Snoring | A | C | 0.414047 | 0.024626263 | 0.004469423 |
| rs10878269 | Snoring | T | C | 0.363891 | 0.039492199 | 0.004568369 |
| rs11008320 | Snoring | G | A | 0.41759 | 0.025212098 | 0.004450439 |
| rs11018488 | Snoring | A | T | 0.626825 | 0.028606739 | 0.004621849 |
| rs11075985 | Snoring | A | C | 0.423353 | 0.02920423 | 0.004445254 |
| rs1108431 | Snoring | T | C | 0.372924 | 0.028251407 | 0.004540558 |
| rs1115535 | Snoring | T | C | 0.726316 | 0.026721146 | 0.004933472 |
| rs11205713 | Snoring | C | T | 0.433438 | 0.02763536 | 0.004557699 |
| rs11570470 | Snoring | T | A | 0.938269 | 0.052377667 | 0.009156707 |
| rs12128472 | Snoring | G | C | 0.076996 | 0.050497731 | 0.00825822 |
| rs12429765 | Snoring | A | G | 0.508276 | 0.029066546 | 0.004418857 |
| rs12603115 | Snoring | C | T | 0.422369 | 0.027158155 | 0.004443583 |
| rs13251292 | Snoring | G | A | 0.410751 | 0.031269846 | 0.004488021 |
| rs138246582 | Snoring | G | A | 0.927629 | 0.047025399 | 0.008670461 |
| rs147114641 | Snoring | A | C | 0.22647 | 0.03137492 | 0.005244623 |
| rs1609721 | Snoring | T | C | 0.600943 | 0.02606619 | 0.00449282 |
| rs17060460 | Snoring | G | A | 0.226742 | 0.029902168 | 0.005246723 |
| rs17151229 | Snoring | C | G | 0.341142 | 0.027298669 | 0.004636376 |
| rs180110 | Snoring | A | G | 0.590431 | 0.03107791 | 0.004457852 |
| rs2049045 | Snoring | G | C | 0.813698 | 0.036431464 | 0.005640108 |
| rs2224195 | Snoring | C | T | 0.454324 | 0.024726494 | 0.004409772 |
| rs2277339 | Snoring | T | G | 0.896315 | 0.041449569 | 0.00720585 |
| rs2307111 | Snoring | T | C | 0.60636 | 0.032864513 | 0.004498734 |
| rs254557 | Snoring | G | T | 0.671051 | 0.029788052 | 0.004676957 |
| rs2648315 | Snoring | T | C | 0.340076 | 0.02574891 | 0.004661744 |
| rs2664299 | Snoring | T | C | 0.579836 | 0.030401013 | 0.004461752 |
| rs2762049 | Snoring | C | G | 0.392255 | 0.029859058 | 0.004501648 |
| rs33998002 | Snoring | T | A | 0.649272 | 0.026372071 | 0.004593652 |
| rs34811474 | Snoring | G | A | 0.768238 | 0.033018011 | 0.00520597 |
| rs4523230 | Snoring | T | A | 0.715337 | 0.03151629 | 0.004883934 |
| rs4744369 | Snoring | A | T | 0.586733 | 0.026347602 | 0.004492134 |
| rs4815897 | Snoring | G | A | 0.188166 | 0.031825042 | 0.005605655 |
| rs4815915 | Snoring | A | G | 0.636593 | 0.027718065 | 0.004584096 |
| rs4836003 | Snoring | G | C | 0.292227 | 0.026389897 | 0.004826769 |
| rs56319902 | Snoring | T | C | 0.224464 | 0.032658565 | 0.005264721 |
| rs592333 | Snoring | G | A | 0.444166 | 0.038104551 | 0.004421599 |
| rs6099273 | Snoring | T | C | 0.251889 | 0.029555963 | 0.005074885 |
| rs67155855 | Snoring | A | G | 0.203363 | 0.032544234 | 0.005630038 |
| rs725861 | Snoring | G | A | 0.188187 | 0.038286289 | 0.005623953 |
| rs72902175 | Snoring | T | C | 0.134669 | 0.050607005 | 0.006437549 |
| rs73030263 | Snoring | T | C | 0.904607 | 0.04719338 | 0.007496518 |
| rs743122 | Snoring | C | T | 0.301317 | 0.02645499 | 0.004785545 |
| rs796527 | Snoring | G | T | 0.453887 | 0.02788009 | 0.004425542 |
| rs8069947 | Snoring | C | T | 0.553965 | 0.028717384 | 0.004421728 |
| rs8081548 | Snoring | T | A | 0.341764 | 0.027032169 | 0.004645375 |
| rs9583546 | Snoring | C | G | 0.632836 | 0.025056415 | 0.0045463 |
| rs9933881 | Snoring | C | T | 0.075167 | 0.046432321 | 0.008370451 |
| rs10102398 | Ease of awakening | G | A | 0.817735 | 0.0123742 | 0.00203489 |
| rs1017168 | Ease of awakening | C | A | 0.644901 | 0.00932908 | 0.00164666 |
| rs10175975 | Ease of awakening | T | C | 0.180126 | 0.0117081 | 0.00205545 |
| rs10518446 | Ease of awakening | C | G | 0.163148 | 0.0196057 | 0.00212626 |
| rs10748432 | Ease of awakening | C | T | 0.505225 | 0.00861758 | 0.00157124 |
| rs10779704 | Ease of awakening | C | A | 0.628308 | 0.00936381 | 0.00163937 |
| rs11075924 | Ease of awakening | C | A | 0.506388 | 0.00979185 | 0.0015726 |
| rs111329469 | Ease of awakening | C | T | 0.272064 | 0.0103466 | 0.00176344 |
| rs112613078 | Ease of awakening | G | A | 0.190679 | 0.0120864 | 0.00199617 |
| rs116131939 | Ease of awakening | T | C | 0.083057 | 0.0194831 | 0.00288046 |
| rs11672103 | Ease of awakening | C | T | 0.445341 | 0.00884873 | 0.00158915 |
| rs11925088 | Ease of awakening | G | T | 0.075923 | 0.0177824 | 0.00310508 |
| rs12044778 | Ease of awakening | G | A | 0.819974 | 0.0128625 | 0.00204776 |
| rs12154082 | Ease of awakening | A | G | 0.135267 | 0.0125278 | 0.00231243 |
| rs12191085 | Ease of awakening | T | C | 0.741331 | 0.0096568 | 0.0018055 |
| rs12227309 | Ease of awakening | T | C | 0.247532 | 0.0120296 | 0.00182016 |
| rs1256112 | Ease of awakening | C | T | 0.441444 | 0.00919503 | 0.00158824 |
| rs12736689 | Ease of awakening | C | T | 0.030392 | 0.0500764 | 0.00458731 |
| rs12752290 | Ease of awakening | C | T | 0.442836 | 0.0117437 | 0.0015862 |
| rs12920020 | Ease of awakening | A | G | 0.603125 | 0.00978382 | 0.00160323 |
| rs13116306 | Ease of awakening | C | T | 0.578692 | 0.0090291 | 0.0016004 |
| rs13155750 | Ease of awakening | A | G | 0.765711 | 0.0129283 | 0.00185133 |
| rs1333536 | Ease of awakening | C | T | 0.614532 | 0.00927893 | 0.00161944 |
| rs1402954 | Ease of awakening | T | C | 0.090485 | 0.0153996 | 0.00273529 |
| rs1420607 | Ease of awakening | A | G | 0.272546 | 0.0118959 | 0.00176829 |
| rs1421085 | Ease of awakening | C | T | 0.402556 | 0.0110303 | 0.0016006 |
| rs145831787 | Ease of awakening | C | T | 0.8864 | 0.0158154 | 0.00247301 |
| rs1588414 | Ease of awakening | G | A | 0.434678 | 0.00961764 | 0.00158422 |
| rs17464772 | Ease of awakening | A | G | 0.353719 | 0.0126243 | 0.00164542 |
| rs177552 | Ease of awakening | T | C | 0.673425 | 0.00995432 | 0.00167611 |
| rs1854558 | Ease of awakening | G | A | 0.73599 | 0.0101881 | 0.00178604 |
| rs2119876 | Ease of awakening | A | G | 0.2138 | 0.0107791 | 0.00191699 |
| rs2216614 | Ease of awakening | G | A | 0.381777 | 0.00899135 | 0.0016224 |
| rs2360802 | Ease of awakening | T | A | 0.225012 | 0.0127378 | 0.001885 |
| rs2653355 | Ease of awakening | C | A | 0.178283 | 0.0232164 | 0.00205538 |
| rs2944804 | Ease of awakening | C | A | 0.313604 | 0.0105655 | 0.00169677 |
| rs2971970 | Ease of awakening | T | G | 0.217952 | 0.0115111 | 0.00190227 |
| rs3120882 | Ease of awakening | T | C | 0.801518 | 0.0131578 | 0.0019656 |
| rs34327448 | Ease of awakening | A | T | 0.671116 | 0.0111845 | 0.00167628 |
| rs34757401 | Ease of awakening | G | A | 0.225948 | 0.0105842 | 0.00189353 |
| rs3760185 | Ease of awakening | C | T | 0.751635 | 0.0115384 | 0.0018381 |
| rs3798519 | Ease of awakening | C | A | 0.179793 | 0.0112914 | 0.00204673 |
| rs3850174 | Ease of awakening | T | A | 0.743133 | 0.00996604 | 0.00181158 |
| rs4395148 | Ease of awakening | A | T | 0.691678 | 0.00992684 | 0.00170421 |
| rs4483990 | Ease of awakening | A | C | 0.842728 | 0.0180339 | 0.0021638 |
| rs45534736 | Ease of awakening | G | C | 0.055523 | 0.019053 | 0.00342385 |
| rs4784655 | Ease of awakening | G | C | 0.678638 | 0.00973889 | 0.00168589 |
| rs4790352 | Ease of awakening | A | G | 0.9180356 | 0.0179696 | 0.00286535 |
| rs4958317 | Ease of awakening | A | G | 0.289292 | 0.0133255 | 0.0017316 |
| rs59563980 | Ease of awakening | T | C | 0.384825 | 0.00920571 | 0.00161951 |
| rs620598 | Ease of awakening | G | A | 0.225504 | 0.0113138 | 0.00188311 |
| rs62080068 | Ease of awakening | T | C | 0.292241 | 0.0098977 | 0.00173081 |
| rs62165148 | Ease of awakening | G | A | 0.369408 | 0.0105772 | 0.00163043 |
| rs62441739 | Ease of awakening | G | C | 0.791451 | 0.0107961 | 0.00193202 |
| rs6676685 | Ease of awakening | A | C | 0.478079 | 0.00941953 | 0.00158173 |
| rs6697589 | Ease of awakening | C | T | 0.705322 | 0.00938184 | 0.00171929 |
| rs67446253 | Ease of awakening | T | C | 0.343607 | 0.0102954 | 0.00166756 |
| rs6745423 | Ease of awakening | T | A | 0.277264 | 0.0124007 | 0.00175444 |
| rs6960360 | Ease of awakening | G | A | 0.600662 | 0.00959479 | 0.00160624 |
| rs698813 | Ease of awakening | G | A | 0.765416 | 0.0142901 | 0.00185324 |
| rs7105482 | Ease of awakening | G | A | 0.393466 | 0.0091166 | 0.00160925 |
| rs7219015 | Ease of awakening | C | T | 0.781223 | 0.0108514 | 0.0019045 |
| rs7222039 | Ease of awakening | T | C | 0.444019 | 0.00920538 | 0.0015792 |
| rs7289389 | Ease of awakening | A | T | 0.758087 | 0.0101174 | 0.00183758 |
| rs72895663 | Ease of awakening | G | A | 0.231857 | 0.0107471 | 0.00185919 |
| rs735119 | Ease of awakening | T | G | 0.272561 | 0.00993866 | 0.00176219 |
| rs73671843 | Ease of awakening | A | G | 0.131766 | 0.0129391 | 0.00235319 |
| rs74643199 | Ease of awakening | A | T | 0.84025 | 0.0123147 | 0.00216809 |
| rs75117727 | Ease of awakening | G | A | 0.482325 | 0.0100754 | 0.00157146 |
| rs7545893 | Ease of awakening | A | C | 0.195483 | 0.0161617 | 0.0019813 |
| rs75650221 | Ease of awakening | T | C | 0.038395 | 0.0341477 | 0.00408745 |
| rs7593335 | Ease of awakening | A | G | 0.28737 | 0.0100368 | 0.00173104 |
| rs76109036 | Ease of awakening | C | T | 0.913504 | 0.0182917 | 0.00279746 |
| rs77556405 | Ease of awakening | A | G | 0.173601 | 0.0172327 | 0.002071 |
| rs77556698 | Ease of awakening | G | T | 0.783428 | 0.0109198 | 0.00191002 |
| rs78054593 | Ease of awakening | C | T | 0.932083 | 0.0172534 | 0.00314124 |
| rs78304176 | Ease of awakening | G | T | 0.935952 | 0.0179395 | 0.00322889 |
| rs7833021 | Ease of awakening | T | C | 0.772702 | 0.0117221 | 0.00187986 |
| rs7927022 | Ease of awakening | T | C | 0.68691 | 0.0124767 | 0.00169214 |
| rs7943634 | Ease of awakening | C | T | 0.690717 | 0.00957038 | 0.00169966 |
| rs80028338 | Ease of awakening | C | A | 0.20575 | 0.0139655 | 0.00199982 |
| rs80261926 | Ease of awakening | T | C | 0.018393 | 0.0388518 | 0.00592782 |
| rs853658 | Ease of awakening | T | A | 0.920805 | 0.0188742 | 0.00315571 |
| rs9309511 | Ease of awakening | A | G | 0.572784 | 0.0132133 | 0.00158578 |
| rs9349502 | Ease of awakening | G | A | 0.307192 | 0.00963544 | 0.00171252 |
| rs9399613 | Ease of awakening | C | T | 0.710841 | 0.0102808 | 0.00174162 |
| rs11123962 | Daytime sleepiness | G | T | 0.446766 | 0.00804433 | 0.00103439 |
| rs11649804 | Daytime sleepiness | C | A | 0.71018 | 0.00697145 | 0.00113373 |
| rs11942333 | Daytime sleepiness | A | G | 0.324202 | 0.00605348 | 0.00110327 |
| rs12140153 | Daytime sleepiness | G | T | 0.904572 | 0.01658 | 0.00179639 |
| rs12153518 | Daytime sleepiness | A | G | 0.47224 | 0.00670287 | 0.00103166 |
| rs13010456 | Daytime sleepiness | A | G | 0.594693 | 0.00774142 | 0.00105125 |
| rs13097760 | Daytime sleepiness | C | A | 0.361304 | 0.00598755 | 0.00107776 |
| rs13135092 | Daytime sleepiness | G | A | 0.082807 | 0.0103303 | 0.0018774 |
| rs147114641 | Daytime sleepiness | C | A | 0.773748 | 0.00787842 | 0.00122983 |
| rs1566362 | Daytime sleepiness | T | C | 0.631731 | 0.00631993 | 0.0010687 |
| rs1601440 | Daytime sleepiness | C | T | 0.278583 | 0.00723315 | 0.00115111 |
| rs17131124 | Daytime sleepiness | G | C | 0.088476 | 0.0111768 | 0.00183679 |
| rs17356118 | Daytime sleepiness | G | A | 0.23121 | 0.00765559 | 0.00122087 |
| rs17763050 | Daytime sleepiness | G | A | 0.77613 | 0.0081203 | 0.00123568 |
| rs1846644 | Daytime sleepiness | C | T | 0.409151 | 0.0113546 | 0.00104777 |
| rs2787120 | Daytime sleepiness | A | G | 0.832602 | 0.00778398 | 0.00137756 |
| rs285793 | Daytime sleepiness | G | A | 0.461246 | 0.00676457 | 0.00103539 |
| rs3122170 | Daytime sleepiness | C | A | 0.231111 | 0.00950345 | 0.00122688 |
| rs4665972 | Daytime sleepiness | T | C | 0.393644 | 0.0066245 | 0.00105853 |
| rs4765939 | Daytime sleepiness | C | G | 0.416728 | 0.00627707 | 0.00104601 |
| rs553314 | Daytime sleepiness | T | C | 0.365253 | 0.00648171 | 0.00107612 |
| rs55818482 | Daytime sleepiness | C | T | 0.215002 | 0.00973113 | 0.00125942 |
| rs55960940 | Daytime sleepiness | T | C | 0.822172 | 0.00762668 | 0.00135164 |
| rs57746981 | Daytime sleepiness | C | T | 0.644468 | 0.00680998 | 0.00107584 |
| rs62519825 | Daytime sleepiness | C | T | 0.11259 | 0.0094794 | 0.00162829 |
| rs67155855 | Daytime sleepiness | G | A | 0.79678 | 0.00733702 | 0.00132004 |
| rs6741951 | Daytime sleepiness | G | A | 0.710788 | 0.00681617 | 0.00114107 |
| rs6897863 | Daytime sleepiness | A | C | 0.584415 | 0.00645716 | 0.00104732 |
| rs6923811 | Daytime sleepiness | T | C | 0.678973 | 0.00677376 | 0.00110514 |
| rs7476897 | Daytime sleepiness | G | A | 0.679434 | 0.00743007 | 0.00110192 |
| rs7598712 | Daytime sleepiness | G | T | 0.555254 | 0.00579387 | 0.00104191 |
| rs7607363 | Daytime sleepiness | G | A | 0.438766 | 0.0060244 | 0.00103841 |
| rs7837226 | Daytime sleepiness | G | A | 0.527148 | 0.00574201 | 0.00103269 |
| rs8015449 | Daytime sleepiness | A | G | 0.538612 | 0.00619154 | 0.00103462 |
| rs825127 | Daytime sleepiness | T | G | 0.530794 | 0.00591296 | 0.00103275 |
| rs843372 | Daytime sleepiness | C | T | 0.230053 | 0.00816368 | 0.00122597 |
| rs886114 | Daytime sleepiness | C | T | 0.357313 | 0.00604278 | 0.00107429 |
| rs960986 | Daytime sleepiness | C | T | 0.636525 | 0.00716034 | 0.00106799 |
| rs9712275 | Daytime sleepiness | T | C | 0.514118 | 0.00588405 | 0.0010319 |
